# Turning Prickly Pear Processing Waste Into Bioactive Ingredients: Fraction‐Specific Drying and Green Extraction of Phenolics, Flavonoids, and Betalains From *Opuntia ficus-indica* (L.) Mill Peel and Pulp

**DOI:** 10.1155/ijfo/7736054

**Published:** 2026-11-15

**Authors:** Thetis Gkogkou, Dimitris Gkavanozis, Patroklos Vareltzis

**Affiliations:** ^1^ Laboratory of Food Technology and Agricultural Industries Department of Chemical Engineering Aristotle University of Thessaloniki Thessaloniki Greece auth.gr

**Keywords:** drying kinetics, extraction, phenolics, prickly pear, response surface methodology, waste valorization

## Abstract

Industrial processing of prickly pear generates significant quantities of peel and pulp waste, which are rich sources of bioactive compounds. Drying and extraction for these waste streams have been reported previously; however, most cases examine only a single fraction under differing experimental conditions. This prevents meaningful comparison of the published optima across fractions. Therefore, this study applied a common framework to both fractions. Peel and pulp were subjected to the same series of experimental conditions including drying temperature and target residual moisture levels, followed by extraction designs with these objectives: (i) to identify the difference in drying and extraction kinetics and overall behavior between the two fractions, (ii) to test the potential of ultrasound applied as predrying step to improve bioactive retention relative to conventional oven drying, and (iii) to determine whether this green solvent approach provides competitive results compared with more aggressive organic solvents for extraction. This study investigated the drying kinetics, how ultrasound pretreatment and final moisture content affect extractability of bioactives and optimized the recovery of phytochemicals. Results can be used to propose a sustainable valorization strategy. Oven drying was performed at four temperatures (40°C, 50°C, 65°C, and 75°C). Drying behavior of the peel and pulp was best described by the two‐term model, whereas for 40°C best‐fitted model was Wang and Singh. Response surface methodology optimization showed each fraction required different extraction conditions. For peel, maximum total phenolic content was achieved with 42% water, 58% ethanol, 60 min extraction, and 0.08 g/mL mass‐to‐solvent ratio, yielding 11.47 ± 0.34 mg GAE/g DW. Maximum total flavonoid content was obtained with 38% water, 62% ethanol, 60 min, and 0.09 g/mL, yielding 2.87 ± 0.20 mg QE/g dw. For pulp, optimized conditions produced 5.99 ± 0.15 mg GAE/g d TPC and 1.44 ± 0.10 mg QE/g dw TFC. All optimal results were obtained with oven drying at 75°C. HPLC identified rutin as the dominant compound. Betacyanin and betaxanthin contents were higher in peel. Overall, peel showed superior phytochemical potential, and high‐temperature drying maintained good bioactive extractability.

## 1. Introduction

Currently the global transition toward a circular bioeconomy has created the necessity of a fundamental shift in how the food industry manages agriculture by‐products. *Opuntia ficus-indica* (L.) Mill., known as prickly pear and hereafter abbreviated OFI, is cultivated worldwide, mostly in arid and semiarid regions, where it thrives due to its drought resistance and valued for its nutritional profile [1, 2]. However, the industrial processing of OFI fruits results in massive quantities of waste, such peel and pulp residues which account for up to 50% of fresh fruit weight [3]. Recent studies have shown that *Opuntia* by‐products can be formulated into high‐value ingredients for food, pharmaceutical, cosmetic and material applications, including natural antioxidants, colorants, and prebiotic fibers [4–6]. That alone emphasizes the importance of developing integrated utilization systems which will also reduce environmental pollution and disposal costs.

Drying those by‐products is the most critical first step because they are high in moisture content. Convective drying is still the most common technology for *Opuntia* fruits, as it is simple and inexpensive. Alternative methods are being explored such as freeze‐drying, which offers excellent preservation of phenolics and color but it is very expensive and time consuming, which limits its suitability for large‐scale by‐product valorization [7]. Notably, a study for OFI fruits reported 504.53 mg GE/100 g for total phenolic content (TPC) [8]. Other methods used are microwave and vacuum‐microwave, where drying times are greatly shortened, but they require more complex equipment and careful control to avoid overheating and degradation [7, 9]. Refractance window is a relatively recent emerging technology, although provides rapid, low temperature drying with good retention of bioactive compounds, has limited industrial availability and higher costs [10]. Over the years, predrying treatments have progressively become major research focus, with numerous techniques applied to fruits and vegetables [11]. Current research trends emphasize the use of ultrasound prior to drying, which results in a drastically reduced drying period [12]. However, some studies showed that this resulted in lower yield in total flavonoid and phenolic content to nonpretreatment samples [13, 14]. Specifically, prickly pear evaluated on pretreatments such as blanching, a citric acid dip, and an ethanol dip [10, 15, 16]. Reports of ultrasound use as a stand‐alone predrying step for OFI peel or pulp appear to be limited. Moreover, solar drying was evaluated on prickly pear peel but it is slow and weather‐dependent and generally leads to poor recovery of phenolics and colorants in comparison with controlled methods [7]. Sun drying reduced the total phenolic and flavonoid contents of OFI fruit by approximately 21% and 62%, respectively, relative to microwave drying of the same material [17]. Based on these variables, the convective drying method, although it relies on extended heat exposure, requires lower investment and allows precise control of temperature and time, facilitating kinetic modeling.

Another aspect that strongly affects extractability of bioactive compounds is moisture content, which controls cell‐wall swelling, matrix structure, and balance between diffusion and degradation phenomena [18, 19]. At moderate levels of residual moisture, water acts as a plasticizer and swelling agent for polysaccharide matrix of cell wall. This induces structural loosening, which improves solvent penetration and diffusion pathways, thus facilitating the release of phenolic compounds and pigments [20]. On the contrary, when tissues are dried to very low moisture levels, the cell wall can become very brittle and enter compact state, in which reduced porosity and mobility prevent the amount of extractable compounds [21]. In the particular case of OFI, the distinct morphological contrast between the anatomical fractions determined their behaviors during extraction. For instance, Ettalibi et al. [22] found that terminating the drying of prickly pear peels from three varieties at 15% moisture yielded significantly higher TPC (17.39–24.09 mg GAE/g DW) than dehydration to 10%. Therefore, for by‐products such as prickly pear peels, selecting an appropriate moisture level rather than simply minimizing moisture can be critical.

In this context, it important to note that *O. ficus*‐*indica* fruit is well known as a rich source of diverse bioactive molecules, including phenolic acids, flavonoids, betalains, and vitamin C, which contribute to its strong antioxidant and health‐promoting properties. Several studies have demonstrated that peels generally contain higher concentrations of total phenolics, betalains, and exhibit better antioxidant activity than the corresponding pulps, whereas seeds provide valuable lipids, suggesting that each fraction of the fruit can be targeted for specific functional ingredients [23, 24]. For instance, the peel contains quantities of flavonoids like rutin (8.19 mg/g) and quercetin (6.05 mg/g) alongside important phenolic acids like chlorogenic and piscidic acid [25, 26]. Together, these phytochemicals indicate potent radical‐scavenging and metal‐chelating capacities neutralizing free radicals and preventing lipid peroxidation [27].

In addition to their phenolic content, *Opuntia* by‐products are valuable because of their rich profile of natural pigments. Both peel and pulp contain abundant amounts of betalains, which are water‐soluble, nitrogen‐containing pigments. They are structurally classified into betacyanins (BC) for purple‐red color and betaxanthins (BX) for yellow‐orange color. Reported contents vary widely with cultivar, tissue, and processing, spanning roughly 140–450 and 45–70 mg/100 g DW, respectively [28–30]. Unlike other natural plant pigments, these specifically exhibit remarkable stability over a wide pH range (3–7) [31, 32].

First, it is necessary to stabilize the extraction substrate (waste material) in order to determine the optimal conditions for maximizing recovery yield for bioactive compounds. Modern analytical chemistry is already rapidly shifting away from toxic petroleum‐based organic solvents. This is in favor of “green” extraction principles, prioritizing environmental and human safety. Appropriate selection, not only of the solvent but also its composition determines both yield and the purity of compounds recovered. For the coextraction of the broad spectrum of bioactive compounds found in prickly pear, such as nonpolar flavonoids, highly polar phenolics and betalains, a binary mixture is highly suggested by existing literature [33]. By adjusting the water‐to‐ethanol ratio, the solvent′s polarity is optimized for phytochemical recovery. In this two‐phase system, water naturally bulks up the dry plant tissues to increase porosity, allowing ethanol to effectively extract phenolic compounds without simultaneously extracting unwanted lipids [34].

Considering these variables, it is concluded that the scientific literature on *Opuntia* valorization has traditionally focused on either single‐fraction processing (peel or pulp alone) or applied predrying treatments without systematically comparing their benefits against simplified baseline methods. Because each fraction has been optimized in isolation and under different experimental designs, the published optima are not directly comparable. The present work addresses this gap by studying both side streams of *O. ficus-indica* processing within a common experimental framework, thereby defining processing conditions that are optimal for the valorization of the two streams simultaneously. Peel and pulp were subjected to the same experimental conditions including drying temperature, target residual moisture levels, and extraction designs with the following objectives: (i) to identify the difference in drying and extraction kinetics and overall behavior between the two fractions, (ii) to test the potential of ultrasound applied as predrying step to improve bioactive retention relative to conventional oven drying, and (iii) to determine whether this green solvent approach provides competitive results compared with more aggressive organic solvents for extraction. This integrated approach provides fraction‐specific processing conditions for converting *Opuntia* residues into phenolic‐, flavonoid‐, and betalain‐rich ingredients using food‐compatible ethanol–water extraction. Our primary object was to first determine optimal final moisture level (after drying) and then evaluate the drying kinetics of the two by‐products of OFI, peel and pulp, over a range of temperatures with oven drying and ultrasonic pretreatment. Despite the recognized biological value of prickly pear waste, significant knowledge gaps remain regarding the distinct mass transfer behaviors of its specific fractions.

## 2. Materials and Methods

### 2.1. Reagents, Solvents, and Standards

Folin–Ciocalteu reagent and aluminum chloride were purchased from Sigma‐Aldrich. Gallic acid (≥ 93.5% purity, Glentham Life Sciences, Corsham, United Kingdom), chlorogenic acid (≥ 97% purity, Sigma‐Aldrich, St. Louis, Missouri, United States), caffeic acid (≥ 99.5%, Sigma‐Aldrich,) St. Louis, Missouri, United States), p‐coumaric acid (99.85% purity, Phytolab, Vestenbergsgreuth, Germany), rutin (98.09% purity, MedChemExpress, Monmouth Junction, New Jersey, United States), ferulic acid (99.92%, MedChemExpress, Monmouth Junction New Jersey, United States), and quercetin (95% purity, Sigma‐Aldrich, St. Louis, Missouri, United States) were used as reference standards. HPLC‐grade water, acetonitrile and formic acid were obtained from Fisher Scientific (Waltham, Massachusetts, United States).

All other reagents were of analytical grade and used as received. Standard stock solutions were prepared in methanol and stored at −20°C, protected from light, until analysis.

### 2.2. Raw Material

Samples of *O. ficus-indica* peels and pulp were sourced from a local processing plant in Crete, Greece. In this facility, fruits are processed primarily for seed oil extraction; thus, the peels and pulp used in this study were collected directly from the production line as separated industrial residues. The samples were sealed in polyethylene bags, transported to the laboratory in frozen state, and stored at −20°C in the dark and stored for 1 week before the drying experiments. All drying and pretreatment conditions were applied to material originating from a single homogenized batch, so that storage time is not confounded with treatment. Before drying, the samples were thawed at 4°C for 12 h.

### 2.3. Compositional Analysis

The composition analysis was carried out according to official AOAC methods [35]. Moisture was determined gravimetrically, by drying the samples using oven TYP‐300 (MELAG, Berlin, Germany) at 105°C (AOAC 925.10). Ash content was measured gravimetrically after incineration of samples in a muffle furnace at high temperature (550°C) overnight (Digital Muffle Furnace, FX‐05, Witeg, Wertheim, Germany; AOAC 942.05). Protein measured by the Kjeldahl method using for digestion Kjeldatherm 8‐block (Gerhardt, Königswinter, Germany) and for distillation VAPODEST (Gerhardt, Königswinter, Germany). The appropriate nitrogen was used for protein conversion factor (AOAC 942.05). Crude fat was quantified by solvent extraction in a Soxhlet apparatus (AOAC 920.39). Total dietary fiber was analyzed using the standard enzymatic–gravimetric procedure (AOAC 978.10).

Total carbohydrate was calculated by difference on a fresh‐weight basis as 100 − (moisture + crude protein + ash + crude fat + crude fiber) (FAO, 2003). Because crude fiber rather than total dietary fiber was subtracted, the value includes soluble dietary fiber alongside soluble sugars, nonfiber polysaccharides, and organic acids and carries the combined uncertainty of the five measured fractions. All determinations were performed in triplicate and were expressed as a percentage of fresh weight.

### 2.4. Drying Experiments

Drying experiments were conducted in a laboratory‐scale oven. The experiments were performed at four distinct air temperatures: 40°C, 50°C, 65°C, and 75°C. Thin‐layer drying experiments were conducted using samples of approximately 5–10 g for both the peel and pulp of OFI, layered in a tray. Moisture loss was monitored by weighing every 20 min using a precision balance until constant weight was achieved. Equilibrium moisture content (*M*_*e*_) was estimated from the drying curves as the average of the final moisture values where the drying rate became practically zero (no further change in sample mass). Experiments were performed in triplicates.

#### 2.4.1. Moisture Level Drying

Samples with the same initial moisture content were placed in the oven in separate, identical preweighed aluminum dishes. At each time point, one dish was removed, equilibrated in a desiccator, and reweighed to determine the mass lost as evaporated water. The drying curves were constructed from these measurements. For the experiments at final moisture contents of 15%, 10%, and 5% (wet basis), samples were removed at a predetermined time taken from the drying curve, and their moisture content was verified by the oven method. Upon reaching their respective targets, the samples were immediately cooled in a desiccator and stored for subsequent extraction.

### 2.5. Kinetic Modeling

The experimental moisture ratio (MR) was calculated using Equation (1):

(1) MR=Mt−MeM0−Me,

where *M*_*t*_, *M*_0_, and *M*_*e*_ are the moisture content at time *t*, initial, and equilibrium, respectively.

The drying curves were fitted to seven semiempirical models and empirical thin layer drying models [43] (Table 1). Modeling was performed using Python (statsmodels library and reduced square).

**Table 1:** Semiempirical models for drying curves.

Model	Equation	Reference
Newton	*M* *R* = exp(−*k* *t*)	[36]
Page	*M* *R* = exp(−*k* *t* ^*n*^)	[37]
Modified page	*M* *R* = exp(−(*k* *t*)^*n*^)	[38]
Henderson and Pabis	*M* *R* = *a*exp(−*k* *t*)	[39]
Logarithmic	*M* *R* = *a*exp(−*k* *t*) + *c*	[40]
Wang and Singh	*M* *R* = 1 + *a* *t* + *b* *t* ^2^	[41]
Two‐term	*M* *R* = *a*exp(−*k*_0_ *t*) + *b*exp(−*k*_1_ *t*)	[42]

Model fitting was restricted to the interval 1.00 > MR > 0.05, which corresponds to the removal of 95% of the evaporable water and marks the practical end of the diffusion‐controlled falling rate period. Below MR = 0.05, the remaining water is mostly bound water held by hydrogen bonds to cell‐wall macromolecules. Its removal is controlled by desorption rather than diffusion, placing it outside the validity of thin layer models. In addition, based on Equation (1) the numerator tends zero as *M*_*t*_ approaches *M*_*e*_ and small errors in the estimated equilibrium, moisture content *M*_*e*_ produce large errors in MR at long drying times. For that reason, the terminal points are the least reliable while carrying the greatest leverage on the fit. The same cut‐off was applied to all temperatures and to both fractions, so the ranking of the models does not depend on where it was placed. On the same basis, drying rates below 0.1 H_2_O kg^−1^ DM h^−1^ were not included to exclude the nonevaporative tail. Drying rate data were smoothed with a Savitzky–Golay filter before differentiation (window length = 5, second‐order polynomial).

### 2.6. Extraction Optimization

A response surface methodology (RSM) (@2026 Minitab LLC) was employed to optimize the extraction of bioactive compounds from the dried peels and pulp. A central composite design (CCD) was utilized to investigate the effect of three independent variables. Optimization was performed separately for each response, so that compound‐class‐ and fraction‐specific optimum conditions could be identified. Each optimization therefore maximized a single individual desirability, and no composite desirability across responses was computed.

The design comprised a face‐centered CCD (*α* = 1) in three factors with a full 2^3^ factorial (eight runs), six axial points at the face centers, and five replicated center points. Because published optima for phenolic recovery from *O. ficus-indica* lie at or beyond 60 min [44–46], the design was extended at a fourth time level of 60 min with a 2^2^ factorial in solvent composition (25% and 75% water) and solid‐to‐solvent ratio (0.033 and 0.100 g/mL) and one run at the center composition, giving 24 runs in total. The complete design matrix and measured responses are given in Table S11. The levels are presented in Table 2 and the complete design matrix in Table S11.

**Table 2:** Independent variables and their experimental levels.

Independent variable	Symbol	Unit	Low	Center	High	Additional level
Extraction time	*X*_1_	min	5	17.5	30	60
Mass ratio	*X*_2_	g/mL	0.033 (1:30)	0.050 (1:20)	0.100 (1:10)	—
Solvent ratio (water %)	*X*_3_	%	25 s	50	75	—

The factor ranges were selected based the values reported for the ethanol‐water extraction of *O. ficus-indica* phenolics [44, 47], with the practical limits of the two matrices. Solvent composition was varied from 25%–75% water, a range that covers the polarity window over the phenolic and flavonoid coextraction reported by existing literature [48, 49]. As well as the solid‐to‐solvent ratio, 1:30–1:10 (g/mL) selected to lie within already applied to OFI [47].

The dependent variables (responses) were TPC and total flavonoid content (TFC). The experimental data were fitted to a second‐order polynomial equation to identify the optimal conditions.

Optimum extraction conditions were located using the response optimizer of the software, separately for each response.

### 2.7. Bioactive Compounds

All extractions for bioactive compounds 1 g of dried sample and 20 mL of 1:1 ethanol–water were added, and mixture was stirred magnetically for 30 min, and then centrifuged for 5 min. For betalains content, the extracts were diluted. The extracts were filtered (Whatman No. 1) and stored at −20°C until analysis.

#### 2.7.1. Antioxidant Capacity Determined by 2,2‐Diphenyl‐1‐Picrylhydrazyl DPPH Radical Scavenging

The DPPH radical‐scavenging activity was measured according to the method of Tepe et al. [50]. A 0.1 mL aliquot of this solution was added to 3.9 mL of a 48.5 *μ*M solution of DPPH in methanol, prepared daily and protected from light. The mixtures were incubated for 30 min in the dark, after which the absorbance of the samples and that of the initial DPPH solution were measured at 517 nm using a uniSPEC 2 UV‐Vis spectrophotometer (LLG Labware, Germany). All determinations were performed in triplicate.

The formula used for calculation was

(2) DPPH%Inhibition=AbsDPPH−AbsSampleAbsDPPH∗100,

where *A* *b* *s*_DPPH_ is the absorption of initial DPPH and *A* *b* *s*_Sample_ is the absorbance of the sample.

#### 2.7.2. TPC

The TPC was determined by the Folin–Ciocalteu method based on the procedure used by Singleton et al. with minor modification. After the extraction mentioned above, 1 mL of the extract was added to 5 mL of 0.2 N Folin–Ciocalteu solution, and after 5 min, another 4 mL of 5% w/v Na_2_CO_3_ solution was added. The samples were incubated at 30°C, and after 30 min, their absorbance was measured at a wavelength of 765 nm using uniSPEC 2 Spectrophotometer UV‐Vis (LLG Labware, Germany). A triplet of solutions was created from each sample. TPC was quantified against a gallic acid calibration curve constructed from five standards over the range 0–100 mg GAE/L, each prepared in triplicate and treated identically to the samples (Figure S1). Extracts were diluted as required to bring their absorbance within the calibrated range. The dilution factor differed between drying treatments and was applied in the calculation of each result. TPC expressed as gallic acid equivalents (mg GAE/g DW).

#### 2.7.3. TFC

The AlCl_3_ method was used to determine the TFC as described by Chang et al. After the extraction mentioned above, 0.5 mL aliquot of the extract was mixed with 1.5 mL of ethanol, 0.1 mL of 10% (w/v) AlCl_3_ solution, 0.1 mL of 1 M potassium acetate solution, and 2.8 mL of distilled water, and the mixture was incubated in the dark for 30 min at room temperature. Al(III) forms acid‐stable chelates with the C‐4 keto group and the C‐3 or C‐5 hydroxyl group of flavones and flavonols, producing a yellow complex with an absorption maximum at 415 nm. The assay therefore responds selectively to flavones and flavonols and does not detect flavanones or flavan‐3‐ols, so that the reported TFC is operationally defined by the method. The absorbance was measured at 415 nm using uniSPEC 2 Spectrophotometer UV‐Vis (LLG Labware, Germany). A triplet of solutions was created from each sample. Extracts were diluted as required to bring their absorbance within the calibrated range. The dilution factor differed between drying treatments and was applied in the calculation of each result. TFC was quantified against a gallic acid calibration curve constructed from five standards over the range 0–100 mg QE/L, each prepared in triplicate and treated identically to the samples (Figure S2). Results were expressed as quercetin equivalents (mg QE/g DW).

#### 2.7.4. Betalains Content

The determination of the BC and BX content was performed spectrophotometrically by measuring the absorption of the extracts at two characteristic wavelengths: 535 nm for BC and 480 nm for BX using the following formula according to Yahia and Mondragon‐Jacobo [51]:

(3) Betalains mg100 g=Α∗DF∗MW∗Vd∗100ε∗Wd∗L,

where *A* is the absorption value at the absorption maximum of 535 nm and 483 nm for BC and BX, respectively; *D* *F* is the dilution factor; *V* *d* is the dried sample solution volume (mL), *W* *d* is the dried sample weight (g); and *L* is the path‐length (1 cm) of the cuvette. The molecular weight (*M* *W*) and molar extinction coefficient (*ε*) of betanin (*M* *W* = 550 g/mol; *ε* = 60,000 L mol^−1^ cm^−1^, and *M* *W* = 308 g/mol; *ε* = 48,000 L mol^−1^ cm^−1^) were applied in order to quantify the BC and BX, respectively. The measurements were performed using a uniSPEC 2 Spectrophotometer UV‐Vis (LLG Labware, Germany), and the results were expressed as mg of pigment per 100 g DW.

### 2.8. HPLC‐DAD Determination of Phenolics and Flavonoids

The identification and quantification of bioactive compounds were performed using a high‐performance liquid chromatography (HPLC, Agilent 1200 series, Germany) system coupled with a diode array detector (DAD, Agilent, 1200 series). Chromatographic separation was achieved on a Kromosil C18 analytical column (250 × 4.6 mm, 5 *μ*m) maintained at 30°C. Mobile phase consisted of two solvents: 0.1% formic acid in water (Solvent A) and 100% acetonitrile (Solvent B). The flow rate was maintained at 1 mL/min with an injection volume of 20 *μ*L. Gradient elution program was applied as follows: 0–3 min, 2% B; 3–20 min, 9% B; 20–22 min, 15% B; 22–25 min, 18% B; 25–33 min, 20% B; 33–37 min, 27% B; 37–45 min, 100% B; 45–51 min, 100% B; 51–51.10 min, 2% B; and 51.10–57 min, 2% B for column re‐equilibration. Chromatograms were recorded with a diode array detector at 280 nm for general phenolics, 320 nm for hydroxycinnamic acids [52], and 366 nm for flavonoids as described elsewhere; [53] These wavelengths were selected to ensure selectivity and sensitivity. Identification of major phenolics was based on comparison of retention times and UV spectra with authentic standards (gallic, chlorogenic, caffeic, p‐coumaric, ferulic acids, rutin, and quercetin curves in Figure S3). The resultsand chromatographs, where LOD and LOQ were calculated using ICH Q2 formulas, can be found in Figures S4 and S5 and Table S1.

### 2.9. Statistical Analysis

The experimental results were analyzed using Minitab (@2026 Minitab LLC., United States) to determine the optimal conditions. Separate analyses were applied to three distinct datasets, considering a *p* value less than 0.05 as statistically significant. The compositional dataset for fresh and pulp (Table 3) was compared between the two fractions by one‐way analysis of variance (ANOVA). Prior to that, a separate three‐way ANOVA was performed using general linear model (GLM) to examine the combined influence and interactions of moisture level, temperature, and process for peel and pulp, for each combination of response (TPC and DPPH). TFC, BC, and BX concentrations were evaluated using two‐way ANOVA at the optimal residual moisture content and process, with drying temperature and fraction as factors. The fitted models were assessed by ANOVA of the second‐order regression, from which the significance of each linear, quadratic and interaction with standard error, *R* ^2^, and adjusted *R* ^2^ statistics were obtained. Tukey′s HSD test was applied post hoc for pairwise comparisons only.

**Table 3:** Compositional analysis of fresh peel and fresh pulp.

Material	Moisture g/100 g FW	Protein g/100 g FW	Ash g/100 g FW	Fat g/100 g FW	C. Fibers g/100 g FW	Carbohydrates (g/100 g FW)
Peel	84.10 ± 0.15^a^	4.45 ± 0.05^a^	1.79 ± 0.06^a^	0	7.05 ± 0.13^a^	2.61^a^
Pulp	88.10 ± 0.10^b^	1.10 ± 0.10^b^	0.37 ± 0.03^b^	0	1.61 ± 0.09^b^	8.82^b^

*Note:* Values are mean ± SD (*n* = 3), expressed on a fresh‐weight basis. Means with different letters within a column indicate a significant difference between peel and pulp (one‐way ANOVA, *p* < 0.05). Carbohydrate calculated by difference as 100 − (moisture + crude protein + ash + crude fat + crude fiber).

## 3. Results

### 3.1. Characterization of Raw Peel and Pulp

Protein, ash, and fiber contents in the peel were significantly higher than in pulp (*p* < 0.05). On the other hand, pulp was richer in carbohydrates and showed elevated moisture content (*p* < 0.05).

### 3.2. Drying Kinetics and Fitting of Mathematical Models

The statistical evaluation, based on the highest coefficient of determination (*R* ^2^), lowest root mean square error (RMSE), and lowest reduced chi‐square (*χ* ^2^) revealed a temperature‐dependent shift in the drying kinetics. For the higher drying temperatures (50°C, 65°C, and 75°C), the two‐term model demonstrated the most accurate fit for both the peel and pulp samples, successfully capturing the rapid initial exponential moisture loss and subsequent slower diffusion phases. However, at the lowest drying temperature of 40°C, the Wang and Singh model emerged as the most suitable. This indicates that at 40°C, moisture removal is significantly slower and more gradual, aligning better with the parabolic empirical nature of the Wang and Singh equation (*M* *R* = 1 + *a* *t* + *b* *t* ^2^) rather than the rapid exponential decay observed at higher thermal gradients. Table 4 demonstrates the best‐fitted model for each temperature of peel and pulp. The rest models′ criteria parameters are available in Table S2).

**Table 4:** Constants and statistical values for best‐fitted models.

Sample	Model	Constants	*R* ^2^	RMSE	*χ* ^2^
40°C Peel	Wang–Singh	*a* = −0.2325, *b* = 0.01299	0.9982	0.0123	0.0001672
40°C Pulp	Wang–Singh	*a* = −0.0925, *b* = −0.00078	0.9962	0.0185	0.0003671
50°C Peel	Two‐term	*k*_0_ = 4.319, *k*_1_ = 0.1236,	0.9977	0.0074	0.0000599
		*a* = 0.7377, *b* = 0.2644			
50°C Pulp	Two‐term	*k*_0_ = 0.0068, *k*_1_ = 0.4408,	0.9911	0.0227	0.0005600
		*a* = 0.0694, *b* = 0.9859			
65°C Peel	Two‐term	*k*_0_ = 0.2145, *k*_1_ = 3.4515,	0.9992	0.0088	0.0001541
		*a* = 0.2217, *b* = 0.7785			
65°C Pulp	Two‐term	*k*_0_ = 0.3775, *k*_1_ = 0.3774,	0.9991	0.0067	0.0000583
		*a* = 0.9729, *b* = 0.0784			
75°C Peel	Two‐term	*k*_0_ = 4.1725, *k*_1_ = 0.1116,	0.9989	0.0072	0.0000679
		*a* = 0.8974, *b* = 0.1040			
75°C Pulp	Two‐term	*k*_0_ = 0.9424, *k*_1_ = 0.1837,	0.9816	0.0365	0.00163154
		*a* = 1.0526, *b* = 0.0184			

The fitting for the model presented in Figures 1 and 2 was performed on data with *M* *R* = 1.0–0.05, covering the full kinetically active drying phase. Drying rate was controlled by temperature, whereas higher temperatures produced shorter drying times. At 75°C, both peel and pulp reached significantly faster than 40°C. However, the pulp required longer drying times than the peel along all the temperatures. Especially at 75°C, pulp required approximately 2 h to reach *M* *R* = 0.1, whereas peel needed only 1 h.

**Figure 1:**
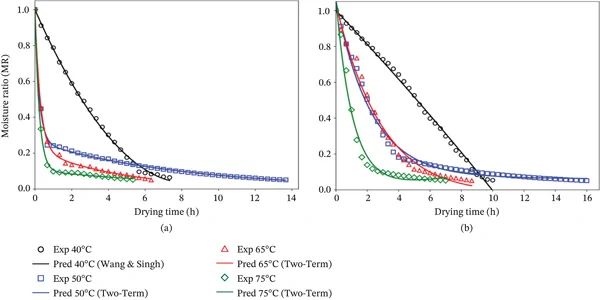
Experimental and predicted moisture ratio (MR) as a function of drying time (h) for prickly pear (a) peel and (b) pulp at different drying temperatures (40°C, 50°C 65°C, and 75°C).

**Figure 2:**
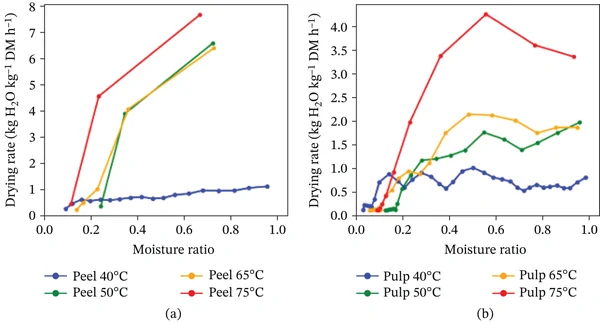
Experimental drying rate (kg H_2_O kg^−1^ DM h^−1^) versus moisture ratio of prickly pear (a) peel and (b) pulp at air drying temperatures of 40°C, 50°C, 65°C, and 75°C.

Experimental data for drying rate as a function of MR, represented in Figure 2, were smoothed using a Savitzky–Golay filter to remove instrumental noise and truncated below 0.1 kg H_2_O kg^−1^ DM h^−1^ to exclude the nonevaporative asymptotic tail [54].

The drying rate for all temperatures 40°C–75°C from Figure 2 is decreased continuously as the MR declined for (Figure 2a) peel, confirming the exclusive presence of the falling rate period that is consistent with the findings discussed above. It is interesting to note that 50°C and 65°C demonstrated overlapping behavior, suggesting a tendency for drying rates to approach equilibrium zone.

### 3.3. Effect of Moisture‐Temperature‐Process on Bioactive Concentration

#### 3.3.1. TPC and DPPH

The effect of different moisture levels, drying temperature, and process on TPC is shown in Figure 3a and on DPPH‐inhibition% in Figure 3b. A three‐factor ANOVA was conducted to ascertain the impact of the primary factors (moisture, temperature, and process) on peel and pulp total polyphenol content.

**Figure 3:**
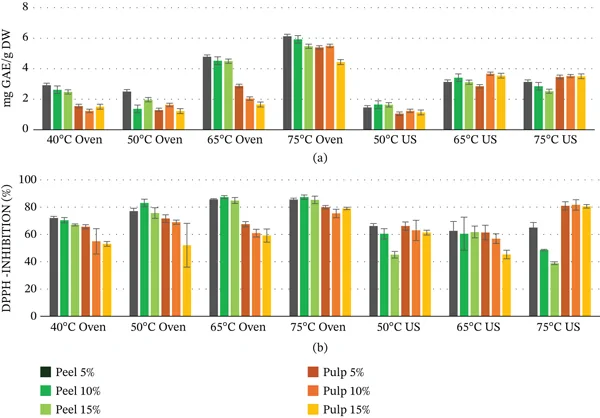
(a) TPC and (b) DPPH‐inhibition % for different moisture levels (5%, 10%, and 15%), temperature (50°C, 65°C, and 75°C), and without and with pretreatment (US) for peel and pulp.

The statistical analysis revealed that all three factors significantly influenced TPC for the peel, with temperature and process demonstrating the highest *F*‐values and thus the strongest effects. The findings of the study indicated significant interactions between moisture and temperature (*p* < 0.001) and between temperature and process (*p* < 0.001) (Table S3). The results demonstrated that the impact of moisture varied according to the drying temperature and that the temperature response differed between the oven and ultrasound treatments. However, the moisture‐process interaction was not significant (*p* = 0.439), and the moisture‐temperature‐process term remained significant (*p* = 0.043). The findings suggest that for the peel, TPC variability is predominantly governed by the main effects of drying temperature and process, whereas moisture exerts a comparatively weaker influence and participates in fewer statistically significant interaction terms. According to the grouping information using Tukey′s method for significance, for all moisture contents, the 75°C drying temperature for the oven‐drying without pretreatment exhibited the highest TPC (Table S4).

For the pulp, temperature was the most significant source of variation in TPC (*F* = 1215.60, *p* < 0.001), whereas several interaction terms also contributed substantially, particularly temperature‐process (*F* = 239.22, *p* < 0.001), moisture‐process (*F* = 27.23, *p* < 0.001), and the moisture‐temperature‐process interaction (*F* = 11.96, *p* < 0.001) (Table S5). This pattern suggests that the effect of each individual factor on pulp TPC is strongly influenced by the levels of the other factors, resulting in a more interaction‐driven response compared with the peel. According to the grouping information using Tukey′s method for significance, the 5% and 10% moisture level at 75°C exhibited the highest TPC content (Table S6).

Ultrasound pretreatment, even though significantly decreased the drying time (Figure S6), it led to lower TPC recoveries. Taking into account the above results, the 10% moisture level at 75°C for the oven‐drying process was selected as optimal.

For both peel and pulp, DPPH scavenging followed similar trend as TPC, confirming that the phenolics are the major contributors to antioxidant capacity. In the peel, all main factors and interactions were significant, with process showing the highest *F*‐value in the DPPH ANOVA (*F* = 593.02, *p* < 0.001), with oven drying displaying superior results in antioxidant activity of extracts compared with pretreatment with US (Table S7). Temperature and moisture content for oven drying process did not exert a significant effect on DPPH (Table S8). In the pulp, the interaction between moisture and process was not significant (*p* > 0.05, Table S9). Temperature was again the dominant effect on DPPH (*F* = 212.67, *p* < 0.001), and the highest antioxidant responses were observed for 75°C treatments, whereas combinations at lower temperatures showed lower DPPH‐inhibition%, consistent with the temperature‐driven trends observed for TPC (Table S10). Also, IC_50_ values for peel dried at 75°C (IC_50_ = 0.62 mg/mL, Figure S7), for oven drying process are the lowest, which can be attributed to the highest TPC content. On the contrary, IC_50_ value for US pretreatment of the pulp are lower compared with the oven‐dried pulp samples. That indicates that a portion of the antioxidant compounds present in the pulp may be thermally degraded or transformed into less active derivatives, leading to an overall deterioration in antioxidant quality relative to ultrasound pretreatment (available in Figure S7). Under the optimal selected conditions (10% final moisture, 75°C drying temperature), the oven‐dried (with no US pretreatment) had TPC 5.93 ± 0.36 and 5.50 ± 0.11 mg GAE/g DW for peel and pulp, respectively.

#### 3.3.2. TFC and Pigments

Samples oven‐dried (without US pretreatment) at a final moisture of 10% were analyzed for TFC and pigment concentration as a function of drying temperature (Figure 4). The highest TFC content was 1.34 ± 0.05 mg QE/g DW for peel and 0.68 ± 0.01 mg QE/g DW for pulp at 75°C drying temperature (Figure 4a). The best yield for pigments was obtained at 75°C, with BX = 27.45 ± 0.08 and BC = 13.11 ± 0.09 mg/100 g DW for peel and BX = 15.41 ± 0.11 and BC = 7.41 ± 0.06 mg/100 g DW for pulp (Figure 4b).

**Figure 4:**
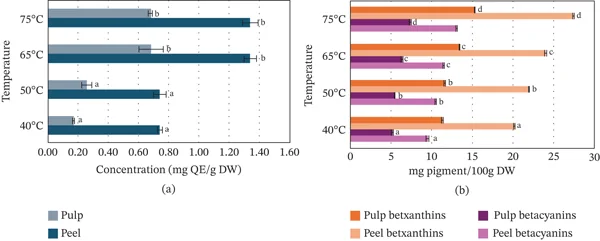
(a) TFC and (b) BC and BX for 10% moisture for different temperatures (40°C, 50°C, 65°C, and 75°C) without pretreatment. Different letters next to bars indicate statistical significance between temperatures within the same tissue, Tukey′s HSD (*p* < 0.05).

### 3.4. Optimization of Extraction Conditions

Extraction and drying kinetics are closely interrelated and determine the final recovery of bioactive compounds [9]. The drying process controls moisture removal rate and temperature exposure, which in return affects the microstructure of the tissues, altering solvent penetration and mass transfer during extraction. Samples were dried at the conditions determined above, and the three extraction parameters were optimized: time, mass‐to‐solvent ratio, and water‐to‐ethanol ratio.

Extraction time was examined at four levels and the other variables at three levels, as reported in Table 2. The outputs were modeled using second‐degree polynomial regression and evaluated using ANOVA to identify statistically significant terms. The combination of DOE with quadratic and interactive terms allowed for the mathematical description of the phenomenon and the optimization of the extraction process. The purpose of this process is to find the maximum possible concentration of phenolics and flavonoids.

#### 3.4.1. Response Surface Model Fitting

The experimental data (Table S11) were fitted to a second‐order polynomial model Equation (4) to describe the relationship between the independent variables and the response variables (TPC and TFC) for peel and pulp extracts:

(4) Y=a+b1X1+b2X2+b3X3+c1X12+c2X22+c3X32+d1X1X2+d2X1X3+d3X2X3,

where *Y* is the predicted response, *a* is the constant (intercept), *b*_1_ – *b*_3_ are the linear coefficients, *c*_1_ – *c*_3_ are the quadratic coefficients, and *d*_1_ – *d*_3_ are the interaction coefficients.

The fitted regression equations for the four responses are as follows:

• Model of peel TPC (mg GAE/g DW)

(5) TPC=−0.200.00271268.730.027636.101672.203.6460.15536.650.0939−X1+X2+X3−×10−6X12−X22−×10−4X32+X1X2−×10−5X1X3+X2X3.

• Model of peel TFC (mg QE/g DW)

(6) FC=0.37130.0378742.260.026633.72249.61.6390.010132.3960.06301+X1+X2−X3−×10−4X12−X22+×10−4X32+X1X2−×10−5X1X3+X2X3.

• Model of pulp TPC (mg GAE/g DW)

(7) TPC=1.59130.1525149.450.069451.966582.49.090.01241.2610.03671+X1−X2+X3−×10−3X12+X22−×10−4X32+X1X2+×10−4X1X3−X2X3.

• Model of pulp TFC (mg QE/g DW)

(8) TFC=−0.43570.00857360.920.037341.133424.23.3090.041523.3700.04272+X1+X2−X3−×10−4X12−X22+×10−4X32+X1X2+×10−5X1X3+X2X3.

Table 5 summarizes the significant important terms and the model adequacy statistics, whereas Table S12 depicts the regression coefficients and *p* values for the second‐order polynomial models. From these tables, it can be concluded that mass‐to‐solvent ratio (g/mL) is the dominant factor for the phenolics in peel, whereas for flavonoids, both time and water content (water/ethanol ratio). On the other hand, TPC for pulp is influenced by time and all three quadratic terms suggesting that time has a clear linear effect while all three factors point to a more complex optimum region. TFC indicates that time is not significant for this response, and mass‐to‐solvent ratio and water % (water‐to‐ethanol ratio) are key drivers. The RSM models that were fitted demonstrated satisfactory predictive capability for all responses, as evidenced by the high *R* ^2^ and adjusted *R* ^2^ values documented in Table 5. The close agreement between these statistics, in conjunction with the relatively low standard errors and the inclusion of only statistically significant linear and quadratic terms (*p* < 0.05), serves to confirm that the models are adequately specified and consistent with the trends observed in the response‐surface plots (Tables S13–S16). Models were also validated using conditions within the examined ranges, which fell into two categories. Confirmation runs were set at the conditions predicted by the RSM for each response and fraction and were used to verify the optimization itself. The conditions for the external runs were chosen to cover both the high‐ and low‐response areas of each surface, rather than being assigned at the optimum. All validation extractions were performed in triplicate on separate portions of the same dried material used for the design. Predicted and experimental values and the corresponding statistics are given in Tables S17–S19).

**Table 5:** Summary of model adequacy statistics for TPC and TFC of peel and pulp.

Response	*R* ^2^	*R* ^2^(adj)	Standard error	Significant terms (*p* < 0.05)
Peel TPC	0.953	0.910	0.469	*X*_2_, $X_{2}^{2}$
Peel TFC	0.903	0.841	0.187	*X*_1_, *X*_3_, $X_{1}^{2}$, $X_{3}^{2}$
Pulp TPC	0.873	0.791	0.561	*X*_1_, $X_{1}^{2}$, $X_{2}^{2}$, $X_{3}^{2}$
Pulp TFC	0.868	0.785	0.121	*X*_2_, *X*_3_, $X_{2}^{2}$, $X_{3}^{2}$

Abbreviations: *X*_1_, time (min); *X*_2_, mass ratio (g/mL); *X*_3_, water (%).

The response surface plots illustrating the combined effects of the independent variables on TPC and TFC are presented in Figure 5 for peel and pulp. Each figure depicts the pairwise interactions: (a) water % vs. time, (b) mass ratio vs. time, and (c) mass ratio vs. water % (water‐to‐ethanol ratio), with the third variable held at its center‐point value.

**Figure 5:**
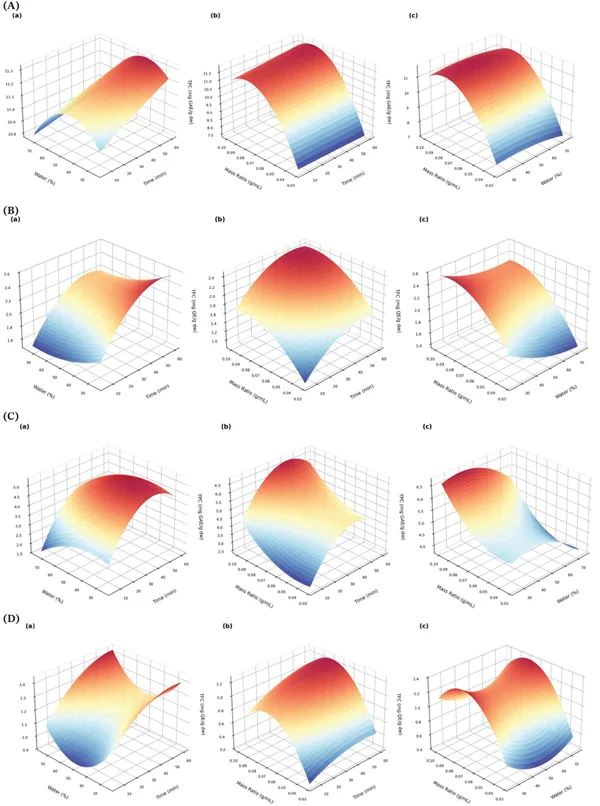
Surface plot showing the effect of (Aa) water concentration vs. extraction time, (Ab) mass‐to‐solvent ratio (g/mL) vs. extraction time, and (Ac) mass‐to‐solvent ratio (g/mL) vs. water concentration for peel extracts for TPC. (Ba) water concentration vs. extraction time, (Bb) mass‐to‐solvent ratio (g/mL) vs. extraction time, and (Bc) mass‐to‐solvent ratio (g/mL) vs. water concentration for peel extracts for TFC. (Ca) mass‐to‐solvent ratio (g/mL) vs. extraction time, (Cb) mass‐to‐solvent ratio (g/mL) vs. water concentration, and (Cc) for pulp extracts for TPC. (Da) water concentration vs. extraction time, (Db) mass‐to‐solvent ratio (g/mL) vs. extraction time, and (Dc) mass‐to‐solvent ratio (g/mL) vs. water concentration for pulp extracts for TFC. The red and blue colors demonstrate the highest and lowest values, respectively.

The optimal conditions for peel TPC (11.47 ± 0.34 mg GAE/g DW) were obtained at 60 min, a mass/solvent ratio of 0.08 g/mL, and 42% water and 58% ethanol. As discussed above, mass‐to‐solvent ratio was the only statistically significant parameter. From Figure 5A, this is visually confirmed by the parabolic relationship observed in the surface plots, where mass transfer resistance and solvent saturation limit yields at ratios deviating from 0.08 g/mL.

From Figure 5B, it is worth mentioning that although the mass‐to‐solvent ratio surface displayed a slight visual curvature, it was not statistically significant parameter for TFC recovery. The maximum experimental TFC yield from the peel (2.87 ± 0.20 mg QE/g DW), closely matching the predicted (2.71 mg QE/g DW), corresponds to an extraction time of 60 min, a mass‐to‐solvent ratio of 0.09 g/mL, and solvent composition of 38% water to 62% ethanol.

For pulp results, optimal phenolic yield from the pulp (5.99 ± 0.15 mg GAE/g DW) is reached using a higher mass‐to‐solvent ratio of 0.10 g/mL, a 38% water mixture, and a 45 min extraction time. Multivariable quadratic significance is demonstrated by the parabolic shape in all the plots in Figure 5Ca–c. This means that extraction efficiency reaches a specific saturation point for all three parameters. Deviating from these optimal conditions causes yield to drop.

The response surface plots for pulp TFC indicate that extraction is primarily governed by solvent composition and mass‐to‐solvent ratio rather than extraction time. The relatively flat response along the time axis in Figure 5Da,b shows that extending extraction time does not substantially increase TFC. The pronounced curvature in Plot c highlights the strong influence of both water–ethanol ratio and mass‐to‐solvent ratio on flavonoid yield. Optimal recovery achieved using mass ratio of 0.08 g/mL paired with 25% water and 75% ethanol and extraction time of 43 min (1.44 ± 0.10 mg QE/g DW).

The optimum conditions predicted for each response were verified experimentally in triplicate (Table 6). Deviations between predicted and measured values ranged from −8.4% to +5.9%, indicating that the fitted models describe the response surfaces adequately within the experimental region and show no systematic bias.

**Table 6:** Predicted and Experimental results of each fraction and response.

Response	Fraction	Predicted	Experimental (± SD, *n* = 3)	S. deviation	Conditions
TPC	Peel	12.44 mg/g	11.47 ± 0.34 mg/g	−7.8%	0.08 g/mL, 58% ethanol, 60 min
TPC	Pulp	6.54 mg/g	5.99 ± 0.15 mg/g	−8.4%	0.09 g/mL, 62% ethanol, 60 min
TFC	Peel	2.71 mg/g	2.87 ± 0.20 mg/g	+5.9%	0.10 g/mL, 62% ethanol, 45 min
TFC	Pulp	1.39 mg/g	1.44 ± 0.10 mg/g	+3.6%	0.08 g/mL, 75% ethanol, 43 min

## 4. Discussion

### 4.1. Compositional Analysis of Peel and Pulp

Most previous studies focus on characterization of dried peel or the stabilized ingredient that will be used in food products [24, 55]. In this study, fresh peel and pulp were characterized to establish the initial matrix of each fraction before convective drying. Given that peel and pulp differ in protein, ash, and fiber content, the compositional profile provides a framework for understanding the effect of matrix composition on the drying kinetics and differences in antioxidant and pigments content after drying.

Both showed high moisture content as expected because peel behaves as a water storage tissue and not only just as a dry protective skin [56]. Fresh peel′s fiber content was higher in comparison with other literature findings for *O. ficus-indica* cultivars and significantly less in ash content [57, 58]. Moisture content of pulp was higher than the one reported by Medina et al. [59] but lower in fibers. These differences are due to ripeness or growing conditions that affect the composition for each part of the fruit.

Additionally, fresh pulp revealed a notably higher carbohydrate content than peel. This is attributed to the physiological role of the tissues during ripening process. In that stage, pulp accumulates high concentrations of monosaccharides that impact the characteristic sweetness of the edible fraction [26]. In contrast, the peel dominated by polysaccharides and dietary fibers, serving a role as protective barrier against environmental stress [60].

### 4.2. Drying Kinetics and Mathematically Fitting Model

Figure 1 demonstrates a steep initial moisture loss for both (a) peel and (b) pulp for temperatures 50°C–75°C reflecting the rapid removal of free water at high moisture contents, followed by a progressive slowing of the drying rate as moisture content decreased.

This two‐phase behavior within the falling rate period has been reported specifically for OFI peel by Panza et al. [61], who identified a fast initial dehydration stage governed by surface evaporation and a slower second stage limited by internal water diffusion. However, at 40°C, this distinction was less evident, as the low thermal driving force produced a more gradual and nearly uniform moisture decline throughout the entire drying process, with no pronounced fast initial stage identifiable in either peel or pulp. No constant rate period is observed; drying occurred exclusively in the falling rate period, which indicates that controlling mechanism of mass transfer is internal moisture diffusion. The Wang and Singh model was superior at 40°C, indicating a transition from exponential to parabolic moisture decline. Notably, the pulp required approximately twice the drying time of the peel at the highest temperature examined. This difference can be directly connected to the structural matrix of each fraction. In contrast, the pulp is rich in sugars, characteristic of OFI fruit, which creates resistance [62]. Also, it is revealed that the pulp on a dry basis holds 7.40 kg H_2_O kg^−1^ DM against 5.29 for the peel, 40% more, before any question of transport arises. A study found the same in shea fruit, where the peel dried faster than the pulp even though the pulp had the higher effective diffusivity. Thus, that indicates drying time follows the mass of water to be removed [63]. The two fractions also differ in what their dry matter is made of. The peel carried 4.4 times more crude fiber (7.05 ± 0.13 against 1.61 ± 0.09 g/100 g f.w.), contributing to a relatively open pore network that allows surface moisture to evaporate quickly. The pulp carried more than three times the carbohydrate (8.82 against 2.61 g/100 g f.w.), mostly low‐molecular‐weight sugars. Sugar‐rich tissue is known to transport water more slowly because of the microstructure raises internal resistance and favors crust formation at the surface [64]. As drying proceeds, the sugars concentrate further, so the effect strengthens. Mucilage may also influence drying behavior because structural and chemical changes in mucilage during dehydration have been linked to irregularities in the drying rate [65].

Data points below the *M* *R* = 0.05 threshold were excluded because the drying curve enters the equilibrium transition zone and models lose predictive reliability [66]. It is important to note that threshold to *M* *R* = 0.05 corresponds to 95% completion of the evaporable water removal relative to the initial moisture content. Below this point the remaining moisture is primarily bound water that is held by hydrogen bonds to macromolecules such as pectin and mucilage. Mechanisms responsible for the removal of the water at this point are different and are therefore outside the valid range of Fick′s diffusion‐based thin‐layer models [67].

That is consistent with the findings by Lahsasni et al. [67] for *O. ficus*‐*indica* fruit under convective solar drying at 50°C–60°C and Nag and Dash [68] for elephant apple pulp dried at 50°C–80°C, where higher mass transfer rates observed at elevated temperatures and by Medina et al. [65] for OFI cladodes at 45°C–85°C under convective oven drying. Panza et al. [61] identified 70°C as the optimal dehydration temperature for OFI peel based on both process productivity and energy consumption indicators.

From Figure 2, it is demonstrated that the maximum drying rate for the pulp at 75°C is roughly 4.1 kg H_2_O kg^−1^ DW h^−1^, which is about half the rate of the peel. That reflects the greater resistance to moisture because of the matrix of the pulp (high content in sugars), as discussed above. Additionally, the pulp curves presented more fluctuations at intermediate MR values (0.2–0.6) compared with smoother curves of the peel, especially at 50°C and 65°C. This phenomenon reflects the heterogeneous water distribution because of the mucilaginous polysaccharide matrix. Similar irregularities have been reported by Medina et al. [65], who attributed fluctuations in the drying rate curve to structural and chemical alterations in mucilage and cellulose cells of the OFI cladodes during dehydration. As demonstrated by Kahn et al. [69], the transport of intracellular water in plant food materials is governed by the structural integrity of the cell matrix, with denser matrices imposing greater resistance to moisture migration throughout drying.

### 4.3. Effect of Moisture‐Temperature‐Process Interaction on Bioactive Concentration

Results showed that TPC content did not downgrade with the higher temperatures, which aligns with previous work of [10, 45]. In addition, peel′s phenolics and flavonoids content was superior to pulp and aligns with the existing research for *Opuntia* species [34, 70].

This trend for TPC is the result of the concertation effect of drying, as moisture is removed, dry matter content per unit mass increases, having as a result higher concentration of phenolic and flavonoid compounds. Studies support the claim that while at higher temperatures, molecular mobility is increased and helps the extractability of bioactive compounds [23, 46].

Peel samples, which had been dried in an oven at a temperature of 75°C, demonstrated the lowest IC_50_ values, a finding that was consistent with their elevated TPC and high DPPH scavenging capacity. In the peel, this suggests that high‐temperature oven drying enhanced phenolic extractability without significantly compromising antioxidant activity. This is likely due to the rapid water loss and fibrous structure, which restricted exposure to damaging heat. Research conducted on spray‐dried probiotic powders produced from fruit peel residues has indicated that the peel matrix can effectively protect sensitive bioactive substances from high‐temperature processing [71]. The extraction yield of phenolic substances is subject to variation depending on the conditions of the drying process. Furthermore, it has been revealed that the peel structure exerts a significant influence on the bioavailability of these substances [72].

In contrast, analysis of the data demonstrated that pulp samples subjected to ultrasound pretreatment demonstrated lower IC_50_ values in comparison with those of the corresponding oven‐dried samples. This finding suggests that although oven drying at 75°C enhanced phenolic recovery, a proportion of the antioxidant fraction within the pulp may be heat‐sensitive, potentially undergoing degradation or transformation into less active derivatives during the drying process [73].

It can be concluded that slower drying behavior of the pulp leads to longer exposure to heat, which probably explains the greater thermal degradation. However, it is also possible that ultrasound pretreatment may have preserved antioxidant quality by facilitating water removal and reducing heat exposure.

#### 4.3.1. Ultrasound Effect as a Pretreatment

Ultrasound applied as a single predrying treatment accelerated moisture removal but conferred no statistically significant benefit on TPC or antioxidant capacity relative to oven drying. This results line up with the matrix‐dependent inconsistency of ultrasound effects on bioactive retention documented by other literature [74]. On the contrary, Aguilar et al. [10] evaluated ultrasound in combination with refractance window drying for OFI material, but no prior research had assessed it as single predrying step for OFI peel or pulp [15, 16]. Ultimately, this research′s results demonstrate that investing in ultrasound pretreatment cannot be justified to boost the bioactive profile of OFI residues, providing a significant finding with economic importance for the design of the industrial processes.

#### 4.3.2. Effect on Pigments

BX and BC content are presented at Figure 4B, showing that BX values were higher in the peel than the pulp, whereas BC were found in lower concentrations. BX fraction was the dominant betalain fraction in both, which is reasonable for yellow‐orange cultivars of OFI. That profile was established by Yeddes et al. who confirmed that the betalain profile varies with fruit color and yellow‐orange OFI varieties are rich in BX [75].

The content of pigments found in our samples after extraction of the optimized dried raw materials is in alignment with recent literature on the oven‐dried of *Opuntia* peel, which reports peel betalain yields 22.74–59.86 mg/100 g DW depending on the applied temperature and pretreatment [16]. Particularly, it is also consistent with values reported in the literature for yellow‐orange OFI, which range from approximately 15–50 mg/100 g DW for BX and 5–20 mg/100 g DW for BC depending on variety, drying temperature, and extraction method [9, 28, 30].

Although at the highest temperature, yield was the highest, it should be noted that betalains are generally highly thermosensitive. Although 75°C could degrade the pigments, the drying time is short and pigments are retained. Studies on the stability of betalains during the processing of red prickly pear show that prolonging thermal exposure at low temperatures causes higher pigment degradation. The authors concluded that fast reduction of water activity is critical for betalain stability [28, 76]. That suggests that this temperature remains within the stability range for both pigments. Given the modern consumer preferences that tend to rapidly increase to reject synthetic colorants, the recovery of highly stable pigment from prickly pear waste offers an exceptionally sustainable, clean label alternative.

### 4.4. Response Surface Model Fitting

The RSM‐optimized peel TPC (11.47 mg GAE/g DW) falls within the lower range reported for OFI peel in the literature (15.70–27.46 mg GAE/g DW, 35.54 mg GAE/g DW), with differences that are attributed to the more aggressive methanol‐based solvents used in those studies. The optimized peel TFC (2.87 mg QE/g DW) exceeds values reported by Belkhir et al. (2.30 mg QE/g DW) [28] using a fully green ethanol–water solvent system, confirming that both fractions hold real potential for valorization as sources of phenolic and flavonoid‐rich extracts.

Fraction‐specific RSM modeling demonstrated that OFI peel and pulp are governed by entirely different extraction variables. Peel TPC was controlled only by solid‐to‐solvent ratio, whereas peel TFC responded to extraction time and water‐to‐ethanol ratio. On the contrary, pulp TPC was influenced by all quadratic terms, whereas pulp TFC required a high ethanol proportion. This most likely reflects the greater solubility of flavonoid aglycones in ethanol, with reduced hydration of the mucilaginous fraction as a possible additional contribution. Prior RSM studies, on microwave and ultrasound‐assisted extraction on *Opuntia* peel and on OFI peel, were confined to a single fraction [3, 77].

Model performance on the independent validation set was lower than the fit statistics of Table 5, and more so for pulp than for peel, the validation *R* ^2^ fell from 0.953 to 0.807 for peel TPC and from 0.873 to 0.737 for pulp TPC. Part of this reduction is structural rather than anomalous. Three factors may contribute to that. Pulp responses are roughly half the magnitude of the peel responses, whereas assay repeatability is comparable in absolute terms, so the same analytical error accounts for a larger share of the pulp signal. The pulp is also the more heterogeneous material, as the greater scatter in its drying‐rate curves at intermediate MRs already indicates. Finally, the pulp surfaces are more sharply curved, which means all three quadratic terms are significant for pulp TPC.

Systematic error was negligible throughout. In every case, the bias is several times smaller than the corresponding random error, indicating that the residual error is random rather than systematic. The complete validation dataset is given in Table S19 and the metric definitions in the supplementary material (Table S1, Table S2, Table S3, Table S4, Table S5).

Overall, this work provides independently developed RSM extraction models for both peel and pulp of OFI using oven green solvent extraction, demonstrating that parameters optimized for one fraction are not transferable to the other.

A research study has been conducted which used a methodology combining RSM with the extraction of various fruit fractions. This study found that the peels and pulps exhibited distinct extraction profiles. A study was conducted on *Hylocereus undatus* (white dragon fruit) and concluded that the peel and pulp require different liquid‐to‐solid ratios [78], indicating that fraction‐specific conditions are required in other cactus fruits as well. Concurrently, RSM‐based research on mandarin peel extraction identified distinct optimization behaviors, with extraction time, solvent composition (acetone/water ratio), and solvent/solid ratio, highlighted as crucial variables, tailored to peel composition. [79].

The conclusion that the control of peel TPC is exclusively governed by the solid‐to‐solvent ratio is attributable to the structural disparities between peels and pulps. A study of pectin extraction from citrus and other sources revealed that the material‐to‐liquor ratio exerts a substantial influence on the yield and quality of extraction [80]. Peel fractions, in particular, demonstrated a notable responsiveness to this parameter. Moreover, research conducted on the extraction of flavonoids from diverse plant peels has repeatedly demonstrated that the variables of “ethanol concentration” and “extraction time”—along with their interplay—exert a substantial influence on the overall recovery of flavonoid content [81].

The findings indicate that the TPC of pulp is influenced by all quadratic terms, which is indicative of the complex polysaccharide matrix present in pulp tissues. As demonstrated by recent research in this field, fruit pulp samples contain higher levels of matrix polysaccharides [74]. This finding suggests that multiple interaction effects may be significant in RSM models. A comprehensive research into the extraction of prickly pear (*O. ficus-indica*) mucilage showed that mucilage from the mesocarp–endocarp is readily precipitated from aqueous solution by ethanol, a property exploited in its isolation [82]. TFC for pulp was maximized at the highest ethanol fraction examined, which is consistent with reduced mucilage hydration under these conditions, although the greater solubility of flavonoid aglycones in ethanol offers a simpler explanation, as mucilage was not determined in the present work.

It is evident that the divergent extraction parameters observed between the peel and the pulp reflect fundamental differences in their composition. A study on fruit by‐products reveals that peel fractions are rich in phenolic compounds, concentrated in the epidermis, whereas pulp contains higher levels of polysaccharides and mucilage compounds. This finding leads to the conclusion that the solid‐to‐solvent ratio alone controls peel extraction, whereas multiple interaction effects govern pulp extraction [5].

## 5. Conclusions

The present study demonstrates that peel and pulp of *O. ficus-indica* processing waste residues should not be treated as a homogeneous waste stream. Their distinct composition is accompanied by different drying kinetics and extraction response, which leads to fraction‐specific recovery conditions. An industrial simple route for recovering phenolics, flavonoids, and betalain pigments, particularly from the peel fraction, was determined, consisting of controlled oven drying at 75°C, 10% final moisture, and a binary mixture of ethanol–water. Another important conclusion is that ultrasound is not recommended as means of assisting the drying process when the objective is phytochemical valorization.

Alongside this, priority future work must also focus on LC–MS/MS confirmation of the tentatively identified isorhamnetin glycoside in the peel and on detailed optimization and LC–MS‐based profiling of the betalain pigment fraction and conducting pilot‐scale trials to ensure extraction reproducibility at an industrial scale. Simultaneously, pilot‐scale trials are required to confirm the reproducibility and robustness of the proposed phenolic and pigment extraction conditions under industrially relevant processing scenarios.

## Author Contributions

**Thetis Gkogkou:** conceptualization, methodology, investigation, formal analysis, data curation, writing – original draft, visualization. **Dimitris Gkavanozis:** formal analysis, writing – original draft. **Patroklos Vareltzis:** conceptualization, supervision, funding acquisition, project administration, resources, writing – review and editing.

## Funding

This study was supported by the Ministry of National Education and Religious Affairs (10.13039/501100006408, YP1TA‐0557915).

## Conflicts of Interest

The authors declare no conflicts of interest.

## Supporting information


**Supporting Information** Additional supporting information can be found online in the Supporting Information section. Standard curves for TPC, TFC and individual phenolics, analytical results of the criteria parameters for the drying models fitting, IC_50_ values, three‐way ANOVA tables from statistical analysis, and typical chromatographs from HPLC analyses are given in the supplementary material.

## Data Availability

The data that support the findings of this study are available from the corresponding author upon reasonable request.
