# Supplementary material for: Turning Prickly Pear Processing Waste Into Bioactive Ingredients: Fraction‐Specific Drying and Green Extraction of Phenolics, Flavonoids, and Betalains From Opuntia ficus-indica (L.) Mill Peel and Pulp
Source: Int J Food Sci. 2026 Nov 15;2026:7736054. doi: 10.1155/ijfo/7736054 (PMC13770290; doi:10.1155/ijfo/7736054)
Supplement: Supplementary file 1 [file IJFO-2026-7736054-s001.docx]

| Concentration (mg GAE / Kg) | Absorbance |
| --- | --- |
| 0 | 0 |
| 25 | 0.190333 |
| 50 | 0.540333 |
| 75 | 0.780667 |
| 100 | 1.02 |

**Figure S1**. Standard Curve Folin for TPC

Standard curve Quercetin

| Concentration (mg QE/ Kg) | Absorbance |
| --- | --- |
| 0 | 0 |
| 10 | 0.091 |
| 25 | 0.203 |
| 50 | 0.407667 |
| 75 | 0.692 |
| 100 | 0.932333 |

**Figure S2**. Standard Curve for TFC

A

B

E

C

D

G

F

| Concentration (mg/Kg) | Area |
| --- | --- |
| 10 | 180 |
| 20 | 384.8 |
| 50 | 1145 |
| 70 | 1641 |
| 100 | 2695 |

| Concentration (mg/Kg) Ttle | Area |
| --- | --- |
| 10 | 494.8 |
| 20 | 874.4 |
| 50 | 2632 |
| 70 | 3898 |
| 100 | 5816 |

| Concentration (mg/Kg) | Area |
| --- | --- |
| 10 | 994.8 |
| 20 | 2100 |
| 50 | 5513.5 |
| 70 | 7792 |
| 100 | 11000 |

| Concentration (mg/Kg) | Area |
| --- | --- |
| 10 | 1173 |
| 20 | 2438 |
| 50 | 6447 |
| 70 | 9115 |
| 100 | 12900.8 |

| Concentration (mg/Kg) | Area |
| --- | --- |
| 10 | 251.8 |
| 20 | 480.7 |
| 50 | 1480.9 |
| 70 | 2001.9 |
| 100 | 2847.6 |

| Concentration (mg/Kg) | Area |
| --- | --- |
| 10 | 896 |
| 20 | 1585.8 |
| 50 | 4668 |
| 70 | 7152.7 |
| 100 | 10257.8 |

| Concentration (mg/Kg) | Area |
| --- | --- |
| 10 | 708.5 |
| 20 | 1599.8 |
| 50 | 3989 |
| 70 | 5863.5 |
| 100 | 8339 |

***Figure S3.*** *Standard curves for HPLC stds, (A) gallic acid, (B) caffeic acid, (C) chlorogenic acid, (D) p-coumaric, ( E ) Rutin, (F) Ferulic acid, (G) Quercetin*

**Figure S3**. Standard curves for HPLC stds, (A) gallic acid, (B) caffeic acid, (C) chlorogenic acid, (D) p-coumaric, ( E ) Rutin, (F) Ferulic acid, (G) Quercetin

mAU

|  | Intensity (mAU) |
| --- | --- |
| 5.003 | 4.69637 |
| 5.00633 | 4.69732 |
| 5.00967 | 4.69637 |
| 5.013 | 4.69398 |
| 5.01633 | 4.69923 |
| 5.01967 | 4.7121 |
| 5.023 | 4.72546 |
| 5.02633 | 4.73022 |
| 5.02967 | 4.73213 |
| 5.033 | 4.73642 |
| 5.03633 | 4.73928 |
| 5.03967 | 4.74262 |
| 5.043 | 4.74834 |
| 5.04633 | 4.76217 |
| 5.04967 | 4.77791 |
| 5.053 | 4.78888 |
| 5.05633 | 4.79507 |
| 5.05967 | 4.79889 |
| 5.063 | 4.80604 |
| 5.06633 | 4.80795 |
| 5.06967 | 4.807 |
| 5.073 | 4.80461 |
| 5.07633 | 4.80747 |
| 5.07967 | 4.81462 |
| 5.083 | 4.82225 |
| 5.08633 | 4.83513 |
| 5.08967 | 4.84896 |
| 5.093 | 4.86517 |
| 5.09633 | 4.87661 |
| 5.09967 | 4.88472 |
| 5.103 | 4.88758 |
| 5.10633 | 4.88853 |
| 5.10967 | 4.89235 |
| 5.113 | 4.89616 |
| 5.11633 | 4.89759 |
| 5.11967 | 4.89855 |
| 5.123 | 4.90522 |
| 5.12633 | 4.91571 |
| 5.12967 | 4.92811 |
| 5.133 | 4.94528 |
| 5.13633 | 4.96864 |
| 5.13967 | 4.98962 |
| 5.143 | 5.00536 |
| 5.14633 | 5.01633 |
| 5.14967 | 5.02443 |
| 5.153 | 5.02872 |
| 5.15633 | 5.03254 |
| 5.15967 | 5.04398 |
| 5.163 | 5.0602 |
| 5.16633 | 5.07593 |
| 5.16967 | 5.0869 |
| 5.173 | 5.09739 |
| 5.17633 | 5.10836 |
| 5.17967 | 5.11932 |
| 5.183 | 5.13029 |
| 5.18633 | 5.14221 |
| 5.18967 | 5.15842 |
| 5.193 | 5.17654 |
| 5.19633 | 5.19705 |
| 5.19967 | 5.21326 |
| 5.203 | 5.2247 |
| 5.20633 | 5.23186 |
| 5.20967 | 5.24044 |
| 5.213 | 5.25284 |
| 5.21633 | 5.26094 |
| 5.21967 | 5.27 |
| 5.223 | 5.28526 |
| 5.22633 | 5.31101 |
| 5.22967 | 5.33438 |
| 5.233 | 5.35059 |
| 5.23633 | 5.36394 |
| 5.23967 | 5.37729 |
| 5.243 | 5.39112 |
| 5.24633 | 5.40495 |
| 5.24967 | 5.42402 |
| 5.253 | 5.44214 |
| 5.25633 | 5.45788 |
| 5.25967 | 5.47409 |
| 5.263 | 5.48983 |
| 5.26633 | 5.49746 |
| 5.26967 | 5.49984 |
| 5.273 | 5.50508 |
| 5.27633 | 5.51987 |
| 5.27967 | 5.53322 |
| 5.283 | 5.54514 |
| 5.28633 | 5.5604 |
| 5.28967 | 5.57804 |
| 5.293 | 5.5933 |
| 5.29633 | 5.60617 |
| 5.29967 | 5.62239 |
| 5.303 | 5.63765 |
| 5.30633 | 5.64957 |
| 5.30967 | 5.66292 |
| 5.313 | 5.68104 |
| 5.31633 | 5.70011 |
| 5.31967 | 5.71489 |
| 5.323 | 5.72968 |
| 5.32633 | 5.74303 |
| 5.32967 | 5.75256 |
| 5.333 | 5.75924 |
| 5.33633 | 5.76639 |
| 5.33967 | 5.77879 |
| 5.343 | 5.79405 |
| 5.34633 | 5.81503 |
| 5.34967 | 5.84173 |
| 5.353 | 5.87034 |
| 5.35633 | 5.89371 |
| 5.35967 | 5.90992 |
| 5.363 | 5.92422 |
| 5.36633 | 5.93996 |
| 5.36967 | 5.95188 |
| 5.373 | 5.96142 |
| 5.37633 | 5.97143 |
| 5.37967 | 5.98574 |
| 5.383 | 6.00052 |
| 5.38633 | 6.01482 |
| 5.38967 | 6.03008 |
| 5.393 | 6.04773 |
| 5.39633 | 6.06775 |
| 5.39967 | 6.0873 |
| 5.403 | 6.10495 |
| 5.40633 | 6.12259 |
| 5.40967 | 6.14262 |
| 5.413 | 6.1636 |
| 5.41633 | 6.18172 |
| 5.41967 | 6.19459 |
| 5.423 | 6.2027 |
| 5.42633 | 6.20413 |
| 5.42967 | 6.20174 |
| 5.433 | 6.19936 |
| 5.43633 | 6.19841 |
| 5.43967 | 6.20079 |
| 5.443 | 6.20556 |
| 5.44633 | 6.21367 |
| 5.44967 | 6.22177 |
| 5.453 | 6.22749 |
| 5.45633 | 6.23322 |
| 5.45967 | 6.23989 |
| 5.463 | 6.24895 |
| 5.46633 | 6.25753 |
| 5.46967 | 6.2604 |
| 5.473 | 6.25801 |
| 5.47633 | 6.25324 |
| 5.47967 | 6.24275 |
| 5.483 | 6.22559 |
| 5.48633 | 6.20747 |
| 5.48967 | 6.19364 |
| 5.493 | 6.18315 |
| 5.49633 | 6.1698 |
| 5.49967 | 6.15835 |
| 5.503 | 6.15358 |
| 5.50633 | 6.14786 |
| 5.50967 | 6.13689 |
| 5.513 | 6.12259 |
| 5.51633 | 6.11162 |
| 5.51967 | 6.10256 |
| 5.523 | 6.08826 |
| 5.52633 | 6.06871 |
| 5.52967 | 6.04963 |
| 5.533 | 6.0339 |
| 5.53633 | 6.0215 |
| 5.53967 | 6.01053 |
| 5.543 | 6.00195 |
| 5.54633 | 5.99909 |
| 5.54967 | 5.99718 |
| 5.553 | 5.99337 |
| 5.55633 | 5.98383 |
| 5.55967 | 5.96905 |
| 5.563 | 5.95856 |
| 5.56633 | 5.95093 |
| 5.56967 | 5.94378 |
| 5.573 | 5.93758 |
| 5.57633 | 5.93042 |
| 5.57967 | 5.9247 |
| 5.583 | 5.91373 |
| 5.58633 | 5.89991 |
| 5.58967 | 5.88799 |
| 5.593 | 5.87511 |
| 5.59633 | 5.86319 |
| 5.59967 | 5.85318 |
| 5.603 | 5.84841 |
| 5.60633 | 5.84364 |
| 5.60967 | 5.83887 |
| 5.613 | 5.83649 |
| 5.61633 | 5.83792 |
| 5.61967 | 5.83839 |
| 5.623 | 5.83553 |
| 5.62633 | 5.82933 |
| 5.62967 | 5.82218 |
| 5.633 | 5.81503 |
| 5.63633 | 5.80502 |
| 5.63967 | 5.79405 |
| 5.643 | 5.7826 |
| 5.64633 | 5.77784 |
| 5.64967 | 5.77641 |
| 5.653 | 5.77736 |
| 5.65633 | 5.77688 |
| 5.65967 | 5.7745 |
| 5.663 | 5.77259 |
| 5.66633 | 5.76687 |

**min**

mAU

|  | Intensity (mAU) |
| --- | --- |
| 5.003 | 0.995159 |
| 5.00633 | 0.987053 |
| 5.00967 | 0.983715 |
| 5.013 | 0.983715 |
| 5.01633 | 0.986576 |
| 5.01967 | 0.990391 |
| 5.023 | 0.995159 |
| 5.02633 | 0.998497 |
| 5.02967 | 0.999928 |
| 5.033 | 0.99659 |
| 5.03633 | 0.991821 |
| 5.03967 | 0.986099 |
| 5.043 | 0.9799 |
| 5.04633 | 0.974655 |
| 5.04967 | 0.971794 |
| 5.053 | 0.973225 |
| 5.05633 | 0.974655 |
| 5.05967 | 0.976086 |
| 5.063 | 0.981808 |
| 5.06633 | 0.988483 |
| 5.06967 | 0.992298 |
| 5.073 | 0.989914 |
| 5.07633 | 0.986099 |
| 5.07967 | 0.983238 |
| 5.083 | 0.979424 |
| 5.08633 | 0.975132 |
| 5.08967 | 0.973225 |
| 5.093 | 0.973225 |
| 5.09633 | 0.971794 |
| 5.09967 | 0.968933 |
| 5.103 | 0.966549 |
| 5.10633 | 0.964642 |
| 5.10967 | 0.962734 |
| 5.113 | 0.964165 |
| 5.11633 | 0.969887 |
| 5.11967 | 0.97847 |
| 5.123 | 0.984192 |
| 5.12633 | 0.986576 |
| 5.12967 | 0.988007 |
| 5.133 | 0.988007 |
| 5.13633 | 0.985622 |
| 5.13967 | 0.981331 |
| 5.143 | 0.978947 |
| 5.14633 | 0.979424 |
| 5.14967 | 0.9799 |
| 5.153 | 0.97847 |
| 5.15633 | 0.977039 |
| 5.15967 | 0.977993 |
| 5.163 | 0.979424 |
| 5.16633 | 0.978947 |
| 5.16967 | 0.97847 |
| 5.173 | 0.97847 |
| 5.17633 | 0.97847 |
| 5.17967 | 0.975609 |
| 5.183 | 0.972748 |
| 5.18633 | 0.972271 |
| 5.18967 | 0.973225 |
| 5.193 | 0.973225 |
| 5.19633 | 0.973701 |
| 5.19967 | 0.974655 |
| 5.203 | 0.976562 |
| 5.20633 | 0.97847 |
| 5.20967 | 0.978947 |
| 5.213 | 0.980854 |
| 5.21633 | 0.980377 |
| 5.21967 | 0.980377 |
| 5.223 | 0.980377 |
| 5.22633 | 0.982761 |
| 5.22967 | 0.983715 |
| 5.233 | 0.983238 |
| 5.23633 | 0.982761 |
| 5.23967 | 0.982761 |
| 5.243 | 0.983238 |
| 5.24633 | 0.983238 |
| 5.24967 | 0.986099 |
| 5.253 | 0.988007 |
| 5.25633 | 0.989914 |
| 5.25967 | 0.989437 |
| 5.263 | 0.98753 |
| 5.26633 | 0.981808 |
| 5.26967 | 0.974655 |
| 5.273 | 0.971317 |
| 5.27633 | 0.971317 |
| 5.27967 | 0.97084 |
| 5.283 | 0.967026 |
| 5.28633 | 0.963211 |
| 5.28967 | 0.96035 |
| 5.293 | 0.96035 |
| 5.29633 | 0.963211 |
| 5.29967 | 0.968933 |
| 5.303 | 0.976562 |
| 5.30633 | 0.984192 |
| 5.30967 | 0.989914 |
| 5.313 | 0.992298 |
| 5.31633 | 0.991821 |
| 5.31967 | 0.990391 |
| 5.323 | 0.991821 |
| 5.32633 | 0.995636 |
| 5.32967 | 0.999928 |
| 5.333 | 1.00088 |
| 5.33633 | 0.999928 |
| 5.33967 | 0.998497 |
| 5.343 | 0.997066 |
| 5.34633 | 0.996113 |
| 5.34967 | 0.995636 |
| 5.353 | 0.997543 |
| 5.35633 | 0.99802 |
| 5.35967 | 0.997066 |
| 5.363 | 0.994682 |
| 5.36633 | 0.993252 |
| 5.36967 | 0.990391 |
| 5.373 | 0.988007 |
| 5.37633 | 0.988483 |
| 5.37967 | 0.991821 |
| 5.383 | 0.994682 |
| 5.38633 | 0.995636 |
| 5.38967 | 0.997066 |
| 5.393 | 1.00136 |
| 5.39633 | 1.00613 |
| 5.39967 | 1.00613 |
| 5.403 | 1.0066 |
| 5.40633 | 1.00803 |
| 5.40967 | 1.00899 |
| 5.413 | 1.0066 |
| 5.41633 | 1.00231 |
| 5.41967 | 1.00183 |
| 5.423 | 1.00231 |
| 5.42633 | 1.00327 |
| 5.42967 | 1.00565 |
| 5.433 | 1.00803 |
| 5.43633 | 1.01185 |
| 5.43967 | 1.01423 |
| 5.443 | 1.01566 |
| 5.44633 | 1.01471 |
| 5.44967 | 1.01185 |
| 5.453 | 1.00946 |
| 5.45633 | 1.00756 |
| 5.45967 | 1.00565 |
| 5.463 | 1.00565 |
| 5.46633 | 1.00756 |
| 5.46967 | 1.01137 |
| 5.473 | 1.01471 |
| 5.47633 | 1.01852 |
| 5.47967 | 1.02043 |
| 5.483 | 1.01852 |
| 5.48633 | 1.01423 |
| 5.48967 | 1.01137 |
| 5.493 | 1.00946 |
| 5.49633 | 1.00803 |
| 5.49967 | 1.0066 |
| 5.503 | 1.00708 |
| 5.50633 | 1.00946 |
| 5.50967 | 1.01089 |
| 5.513 | 1.01089 |
| 5.51633 | 1.01185 |
| 5.51967 | 1.01185 |
| 5.523 | 1.01233 |
| 5.52633 | 1.01233 |
| 5.52967 | 1.01089 |
| 5.533 | 1.00899 |
| 5.53633 | 1.00613 |
| 5.53967 | 1.00708 |
| 5.543 | 1.00851 |
| 5.54633 | 1.01042 |
| 5.54967 | 1.01042 |
| 5.553 | 1.00851 |
| 5.55633 | 1.0066 |
| 5.55967 | 1.00374 |
| 5.563 | 1.00279 |
| 5.56633 | 1.00136 |
| 5.56967 | 1.0004 |
| 5.573 | 1.00374 |
| 5.57633 | 1.00994 |
| 5.57967 | 1.01757 |
| 5.583 | 1.02425 |
| 5.58633 | 1.02806 |
| 5.58967 | 1.03045 |
| 5.593 | 1.02901 |
| 5.59633 | 1.0252 |
| 5.59967 | 1.019 |
| 5.603 | 1.01233 |
| 5.60633 | 1.00803 |
| 5.60967 | 1.00565 |
| 5.613 | 1.00613 |
| 5.61633 | 1.0066 |
| 5.61967 | 1.0047 |
| 5.623 | 1.00183 |
| 5.62633 | 0.99802 |
| 5.62967 | 0.995636 |
| 5.633 | 0.993729 |
| 5.63633 | 0.991344 |
| 5.63967 | 0.988007 |
| 5.643 | 0.983238 |
| 5.64633 | 0.97847 |
| 5.64967 | 0.976562 |
| 5.653 | 0.974178 |
| 5.65633 | 0.972748 |
| 5.65967 | 0.974655 |
| 5.663 | 0.982285 |
| 5.66633 | 0.988007 |

mAU

| min | Intensity (mAU) |
| --- | --- |
| 5.003 | 0.403404 |
| 5.00633 | 0.407696 |
| 5.00967 | 0.415802 |
| 5.013 | 0.422001 |
| 5.01633 | 0.421047 |
| 5.01967 | 0.417709 |
| 5.023 | 0.412464 |
| 5.02633 | 0.406742 |
| 5.02967 | 0.401497 |
| 5.033 | 0.399113 |
| 5.03633 | 0.398159 |
| 5.03967 | 0.397682 |
| 5.043 | 0.395775 |
| 5.04633 | 0.39053 |
| 5.04967 | 0.384808 |
| 5.053 | 0.38147 |
| 5.05633 | 0.384331 |
| 5.05967 | 0.386715 |
| 5.063 | 0.392437 |
| 5.06633 | 0.395775 |
| 5.06967 | 0.395298 |
| 5.073 | 0.391483 |
| 5.07633 | 0.390053 |
| 5.07967 | 0.392437 |
| 5.083 | 0.394821 |
| 5.08633 | 0.39959 |
| 5.08967 | 0.406742 |
| 5.093 | 0.412464 |
| 5.09633 | 0.407219 |
| 5.09967 | 0.395775 |
| 5.103 | 0.383377 |
| 5.10633 | 0.372887 |
| 5.10967 | 0.365257 |
| 5.113 | 0.358582 |
| 5.11633 | 0.359058 |
| 5.11967 | 0.360489 |
| 5.123 | 0.365734 |
| 5.12633 | 0.369549 |
| 5.12967 | 0.370502 |
| 5.133 | 0.372887 |
| 5.13633 | 0.378609 |
| 5.13967 | 0.386715 |
| 5.143 | 0.392914 |
| 5.14633 | 0.397205 |
| 5.14967 | 0.402927 |
| 5.153 | 0.405312 |
| 5.15633 | 0.404358 |
| 5.15967 | 0.407219 |
| 5.163 | 0.41151 |
| 5.16633 | 0.414848 |
| 5.16967 | 0.41151 |
| 5.173 | 0.406265 |
| 5.17633 | 0.399113 |
| 5.17967 | 0.390053 |
| 5.183 | 0.3829 |
| 5.18633 | 0.377178 |
| 5.18967 | 0.374317 |
| 5.193 | 0.370502 |
| 5.19633 | 0.368595 |
| 5.19967 | 0.365734 |
| 5.203 | 0.362396 |
| 5.20633 | 0.363827 |
| 5.20967 | 0.372887 |
| 5.213 | 0.390053 |
| 5.21633 | 0.403881 |
| 5.21967 | 0.410557 |
| 5.223 | 0.411987 |
| 5.22633 | 0.41151 |
| 5.22967 | 0.404358 |
| 5.233 | 0.391483 |
| 5.23633 | 0.378132 |
| 5.23967 | 0.369072 |
| 5.243 | 0.362396 |
| 5.24633 | 0.356674 |
| 5.24967 | 0.358105 |
| 5.253 | 0.36335 |
| 5.25633 | 0.373363 |
| 5.25967 | 0.384331 |
| 5.263 | 0.392914 |
| 5.26633 | 0.395298 |
| 5.26967 | 0.389099 |
| 5.273 | 0.383377 |
| 5.27633 | 0.379086 |
| 5.27967 | 0.374794 |
| 5.283 | 0.370979 |
| 5.28633 | 0.370026 |
| 5.28967 | 0.372887 |
| 5.293 | 0.376225 |
| 5.29633 | 0.376701 |
| 5.29967 | 0.375271 |
| 5.303 | 0.375271 |
| 5.30633 | 0.374317 |
| 5.30967 | 0.375748 |
| 5.313 | 0.377655 |
| 5.31633 | 0.384331 |
| 5.31967 | 0.393867 |
| 5.323 | 0.400543 |
| 5.32633 | 0.405312 |
| 5.32967 | 0.407696 |
| 5.333 | 0.407696 |
| 5.33633 | 0.400543 |
| 5.33967 | 0.386238 |
| 5.343 | 0.375748 |
| 5.34633 | 0.374794 |
| 5.34967 | 0.377655 |
| 5.353 | 0.379562 |
| 5.35633 | 0.38147 |
| 5.35967 | 0.389099 |
| 5.363 | 0.40102 |
| 5.36633 | 0.409603 |
| 5.36967 | 0.408649 |
| 5.373 | 0.401974 |
| 5.37633 | 0.392437 |
| 5.37967 | 0.386715 |
| 5.383 | 0.378609 |
| 5.38633 | 0.375271 |
| 5.38967 | 0.380516 |
| 5.393 | 0.391483 |
| 5.39633 | 0.398636 |
| 5.39967 | 0.394821 |
| 5.403 | 0.389576 |
| 5.40633 | 0.383377 |
| 5.40967 | 0.378609 |
| 5.413 | 0.37384 |
| 5.41633 | 0.377178 |
| 5.41967 | 0.388622 |
| 5.423 | 0.397682 |
| 5.42633 | 0.395775 |
| 5.42967 | 0.389099 |
| 5.433 | 0.386238 |
| 5.43633 | 0.3829 |
| 5.43967 | 0.377178 |
| 5.443 | 0.370502 |
| 5.44633 | 0.370502 |
| 5.44967 | 0.371933 |
| 5.453 | 0.370026 |
| 5.45633 | 0.367641 |
| 5.45967 | 0.36478 |
| 5.463 | 0.367165 |
| 5.46633 | 0.370502 |
| 5.46967 | 0.371933 |
| 5.473 | 0.372887 |
| 5.47633 | 0.375271 |
| 5.47967 | 0.381947 |
| 5.483 | 0.386238 |
| 5.48633 | 0.385284 |
| 5.48967 | 0.386238 |
| 5.493 | 0.389576 |
| 5.49633 | 0.389576 |
| 5.49967 | 0.385761 |
| 5.503 | 0.383854 |
| 5.50633 | 0.386238 |
| 5.50967 | 0.387192 |
| 5.513 | 0.380993 |
| 5.51633 | 0.376701 |
| 5.51967 | 0.376225 |
| 5.523 | 0.378609 |
| 5.52633 | 0.380516 |
| 5.52967 | 0.3829 |
| 5.533 | 0.39053 |
| 5.53633 | 0.40102 |
| 5.53967 | 0.407219 |
| 5.543 | 0.404835 |
| 5.54633 | 0.39959 |
| 5.54967 | 0.40102 |
| 5.553 | 0.406265 |
| 5.55633 | 0.406265 |
| 5.55967 | 0.403881 |
| 5.563 | 0.405788 |
| 5.56633 | 0.41008 |
| 5.56967 | 0.407219 |
| 5.573 | 0.401497 |
| 5.57633 | 0.394821 |
| 5.57967 | 0.393867 |
| 5.583 | 0.39196 |
| 5.58633 | 0.386715 |
| 5.58967 | 0.384808 |
| 5.593 | 0.383377 |
| 5.59633 | 0.386715 |
| 5.59967 | 0.390053 |
| 5.603 | 0.396729 |
| 5.60633 | 0.405788 |
| 5.60967 | 0.411034 |
| 5.613 | 0.413418 |
| 5.61633 | 0.410557 |
| 5.61967 | 0.404358 |
| 5.623 | 0.39959 |
| 5.62633 | 0.397682 |
| 5.62967 | 0.398636 |
| 5.633 | 0.39959 |
| 5.63633 | 0.401974 |
| 5.63967 | 0.404358 |
| 5.643 | 0.40102 |
| 5.64633 | 0.39196 |
| 5.64967 | 0.380993 |
| 5.653 | 0.374317 |
| 5.65633 | 0.370502 |
| 5.65967 | 0.369549 |
| 5.663 | 0.371933 |
| 5.66633 | 0.376225 |

**min**

**366 nm**

.

***Figure S4.*** *Chromatographs of dried peel at 280nm, 320nm, 366nm, (****1****) gallic acid, (****2****) chlorgenic acid, (****3****) caffeic, (****4****) p-coumaric, (****5****) ferulic, (****6****) rutin, (****7****) quercetin*

mAU

| min | Intensity (mAU) |
| --- | --- |
| 5.00033 | 2.04229 |
| 5.00367 | 2.04229 |
| 5.007 | 2.04563 |
| 5.01033 | 2.04563 |
| 5.01367 | 2.0442 |
| 5.017 | 2.04086 |
| 5.02033 | 2.03371 |
| 5.02367 | 2.02656 |
| 5.027 | 2.01941 |
| 5.03033 | 2.0175 |
| 5.03367 | 2.01702 |
| 5.037 | 2.01988 |
| 5.04033 | 2.02322 |
| 5.04367 | 2.02417 |
| 5.047 | 2.02417 |
| 5.05033 | 2.0237 |
| 5.05367 | 2.02465 |
| 5.057 | 2.02465 |
| 5.06033 | 2.02703 |
| 5.06367 | 2.0318 |
| 5.067 | 2.03991 |
| 5.07033 | 2.04945 |
| 5.07367 | 2.05946 |
| 5.077 | 2.06852 |
| 5.08033 | 2.07376 |
| 5.08367 | 2.07233 |
| 5.087 | 2.06709 |
| 5.09033 | 2.06137 |
| 5.09367 | 2.05851 |
| 5.097 | 2.05851 |
| 5.10033 | 2.06137 |
| 5.10367 | 2.06852 |
| 5.107 | 2.07901 |
| 5.11033 | 2.09093 |
| 5.11367 | 2.1019 |
| 5.117 | 2.1081 |
| 5.12033 | 2.10905 |
| 5.12367 | 2.10905 |
| 5.127 | 2.11096 |
| 5.13033 | 2.11096 |
| 5.13367 | 2.10905 |
| 5.137 | 2.11 |
| 5.14033 | 2.11477 |
| 5.14367 | 2.12049 |
| 5.147 | 2.12145 |
| 5.15033 | 2.12336 |
| 5.15367 | 2.12479 |
| 5.157 | 2.13003 |
| 5.16033 | 2.13766 |
| 5.16367 | 2.14672 |
| 5.167 | 2.15626 |
| 5.17033 | 2.16389 |
| 5.17367 | 2.17152 |
| 5.177 | 2.17628 |
| 5.18033 | 2.17962 |
| 5.18367 | 2.18296 |
| 5.187 | 2.18678 |
| 5.19033 | 2.19107 |
| 5.19367 | 2.19822 |
| 5.197 | 2.20728 |
| 5.20033 | 2.21348 |
| 5.20367 | 2.21777 |
| 5.207 | 2.21872 |
| 5.21033 | 2.21777 |
| 5.21367 | 2.21968 |
| 5.217 | 2.22731 |
| 5.22033 | 2.24066 |
| 5.22367 | 2.25449 |
| 5.227 | 2.27118 |
| 5.23033 | 2.29311 |
| 5.23367 | 2.3098 |
| 5.237 | 2.31361 |
| 5.24033 | 2.3098 |
| 5.24367 | 2.30837 |
| 5.247 | 2.31314 |
| 5.25033 | 2.31647 |
| 5.25367 | 2.32124 |
| 5.257 | 2.32697 |
| 5.26033 | 2.33507 |
| 5.26367 | 2.34461 |
| 5.267 | 2.35462 |
| 5.27033 | 2.36177 |
| 5.27367 | 2.36893 |
| 5.277 | 2.37799 |
| 5.28033 | 2.39038 |
| 5.28367 | 2.40088 |
| 5.287 | 2.40326 |
| 5.29033 | 2.40326 |
| 5.29367 | 2.40421 |
| 5.297 | 2.41041 |
| 5.30033 | 2.41709 |
| 5.30367 | 2.42233 |
| 5.307 | 2.43235 |
| 5.31033 | 2.44761 |
| 5.31367 | 2.46668 |
| 5.317 | 2.48671 |
| 5.32033 | 2.5034 |
| 5.32367 | 2.52151 |
| 5.327 | 2.53916 |
| 5.33033 | 2.55537 |
| 5.33367 | 2.56348 |
| 5.337 | 2.56443 |
| 5.34033 | 2.56872 |
| 5.34367 | 2.5773 |
| 5.347 | 2.5835 |
| 5.35033 | 2.58493 |
| 5.35367 | 2.58875 |
| 5.357 | 2.60019 |
| 5.36033 | 2.61402 |
| 5.36367 | 2.62928 |
| 5.367 | 2.64502 |
| 5.37033 | 2.66266 |
| 5.37367 | 2.68126 |
| 5.377 | 2.69651 |
| 5.38033 | 2.70462 |
| 5.38367 | 2.70844 |
| 5.387 | 2.71177 |
| 5.39033 | 2.71702 |
| 5.39367 | 2.72131 |
| 5.397 | 2.72846 |
| 5.40033 | 2.74181 |
| 5.40367 | 2.76089 |
| 5.407 | 2.7833 |
| 5.41033 | 2.80571 |
| 5.41367 | 2.83003 |
| 5.417 | 2.85244 |
| 5.42033 | 2.87151 |
| 5.42367 | 2.88391 |
| 5.427 | 2.89249 |
| 5.43033 | 2.90203 |
| 5.43367 | 2.91252 |
| 5.437 | 2.92206 |
| 5.44033 | 2.93159 |
| 5.44367 | 2.94018 |
| 5.447 | 2.95305 |
| 5.45033 | 2.96926 |
| 5.45367 | 2.99168 |
| 5.457 | 3.01743 |
| 5.46033 | 3.0427 |
| 5.46367 | 3.06988 |
| 5.467 | 3.0961 |
| 5.47033 | 3.12042 |
| 5.47367 | 3.13997 |
| 5.477 | 3.15714 |
| 5.48033 | 3.17717 |
| 5.48367 | 3.19767 |
| 5.487 | 3.2177 |
| 5.49033 | 3.23486 |
| 5.49367 | 3.25346 |
| 5.497 | 3.27396 |
| 5.50033 | 3.2959 |
| 5.50367 | 3.3226 |
| 5.507 | 3.35503 |
| 5.51033 | 3.3884 |
| 5.51367 | 3.41988 |
| 5.517 | 3.44801 |
| 5.52033 | 3.47328 |
| 5.52367 | 3.49474 |
| 5.527 | 3.5162 |
| 5.53033 | 3.54385 |
| 5.53367 | 3.57866 |
| 5.537 | 3.62062 |
| 5.54033 | 3.66497 |
| 5.54367 | 3.71218 |
| 5.547 | 3.75652 |
| 5.55033 | 3.79562 |
| 5.55367 | 3.829 |
| 5.557 | 3.85857 |
| 5.56033 | 3.88956 |
| 5.56367 | 3.92151 |
| 5.567 | 3.95536 |
| 5.57033 | 3.9897 |
| 5.57367 | 4.02689 |
| 5.577 | 4.06408 |
| 5.58033 | 4.09508 |
| 5.58367 | 4.12321 |
| 5.587 | 4.15182 |
| 5.59033 | 4.18568 |
| 5.59367 | 4.21762 |
| 5.597 | 4.24719 |
| 5.60033 | 4.27866 |
| 5.60367 | 4.30775 |
| 5.607 | 4.33064 |
| 5.61033 | 4.34589 |
| 5.61367 | 4.36068 |
| 5.617 | 4.37498 |
| 5.62033 | 4.38547 |
| 5.62367 | 4.38976 |
| 5.627 | 4.39167 |
| 5.63033 | 4.39358 |
| 5.63367 | 4.39692 |
| 5.637 | 4.39644 |
| 5.64033 | 4.39405 |
| 5.64367 | 4.38833 |
| 5.647 | 4.38118 |
| 5.65033 | 4.36831 |
| 5.65367 | 4.34685 |
| 5.657 | 4.32014 |
| 5.66033 | 4.2882 |
| 5.66367 | 4.25768 |

**Figure S5**. Chromatograph of dried pulp at 280nm

| **Table S1**. Calibration curve parameters for the quantification of phenolic compounds and results by HPLC-DAD | | | | | | | | | | |
| --- | --- | --- | --- | --- | --- | --- | --- | --- | --- | --- |
| **Compound** | **tR (min)** | **λ (nm)** | **a (slope)** | **b (intercept)** | **R²** | **Intercept Error (σ)** | **LOD (ppm)** | **LOQ (ppm)** | **Mean ± SD** *(µg/g DW)* | **p-value** |
| Gallic acid | 8.58 | 280 | 27.56 | -168.86 | 0.9915 | 88.4 | 10.58 | 32.08 | 110.83 | *—* |
| Chlorogenic acid | 25.30 | 320 | 59.77 | -245.26 | 0.9976 | 102.1 | 5.63 | 17.08 | 161.58 ± 41.87 | 0.0217 ^a^ |
| Caffeic acid | 26.48 | 320 | 113.75 | -103.72 | 0.9998 | 52.7 | 1.56 | 4.72 | 69.75± 20.03 | 0.0232 ^a^ |
| p-Coumaric acid | 30.47 | 320 | 132.67 | -132.13 | 0.9999 | 55.1 | 1.39 | 4.21 | 67.06 ± 7.41 | 0.0036 ^b^ |
| Rutin | 32.02 | 366 | 29.25 | -49.69 | 0.9982 | 43.3 | 4.89 | 14.81 | 944.76 ± 211.60 | 0.0163 ^a^ |
| Ferulic acid | 32.98 | 320 | 106.02 | -389.14 | 0.9976 | 180.7 | 5.62 | 17.04 | 122.00 ± 55.20 | 0.0620 ns |
| Quercetin | 42.11 | 366 | 84.79 | -139.73 | 0.9995 | 64.5 | 2.51 | 7.60 | 61.71 ± 23.58 | 0.0454 ^a^ |
| *Equation: y = ax + b. where y = peak area (mAU·s) and x = concentration (µg/mL)*  *σ taken as the standard error of the intercept from the calibration regression*  *LOD = 3.3*(SE/Slope), LOQ = 10*(σ/S)* | | | | | | | | | | |

| Table S2. Criteria parameters for best fitted for all the models | | | | |
| --- | --- | --- | --- | --- |
| Sample | **Model** | **R^2^** | **Χ^2^** | **RMSE** |
| Peel 40°C | Newton | 0.977939 | 0.001987 | 0.043595 |
| Peel 40°C | Page | 0.994809 | 0.00049 | 0.021146 |
| Peel 40°C | Modified Page | 0.994809 | 0.00049 | 0.021146 |
| Peel 40°C | Henderson & Pabis | 0.982525 | 0.001649 | 0.038799 |
| Peel 40°C | Logarithmic | 0.996388 | 0.000358 | 0.01764 |
| Peel 40°C | Two-Term | 0.982525 | 0.001822 | 0.038799 |
| Peel 40°C | Two-Term Exp | 0.996654 | 0.000332 | 0.016979 |
| Peel 40°C | Wang-Singh | 0.998227 | 0.000167 | 0.012358 |
| Pulp 40°C | Newton | 0.906761 | 0.008752 | 0.092032 |
| Pulp 40°C | Page | 0.98663 | 0.001298 | 0.03485 |
| Pulp 40°C | Modified Page | 0.98663 | 0.001298 | 0.03485 |
| Pulp 40°C | Henderson & Pabis | 0.929067 | 0.006888 | 0.080272 |
| Pulp 40°C | Logarithmic | 0.996101 | 0.000392 | 0.01882 |
| Pulp 40°C | Two-Term | 0.929067 | 0.007398 | 0.080272 |
| Pulp 40°C | Two-Term Exp | 0.995336 | 0.000469 | 0.020583 |
| Pulp 40°C | Wang-Singh | 0.996219 | 0.000367 | 0.018534 |
| Peel 50°C | Newton | 0.460122 | 0.012882 | 0.112139 |
| Peel 50°C | Page | 0.986017 | 0.000342 | 0.018047 |
| Peel 50°C | Modified Page | 0.986017 | 0.000342 | 0.018047 |
| Peel 50°C | Henderson & Pabis | 0.619395 | 0.009308 | 0.094155 |
| Peel 50°C | Logarithmic | 0.895399 | 0.002624 | 0.04936 |
| Peel 50°C | Two-Term | 0.997673 | 5.99E-05 | 0.007362 |
| Peel 50°C | Two-Term Exp | 0.460122 | 0.013542 | 0.112139 |
| Peel 50°C | Wang-Singh | -1.0477 | 0.050081 | 0.218394 |
| Pulp 50°C | Newton | 0.963823 | 0.002124 | 0.045612 |
| Pulp 50°C | Page | 0.969175 | 0.001848 | 0.042104 |
| Pulp 50°C | Modified Page | 0.969175 | 0.001848 | 0.042104 |
| Pulp 50°C | Henderson & Pabis | 0.963838 | 0.002168 | 0.045603 |
| Pulp 50°C | Logarithmic | 0.991044 | 0.000549 | 0.022694 |
| Pulp 50°C | Two-Term | 0.991057 | 0.00056 | 0.022679 |
| Pulp 50°C | Two-Term Exp | 0.988748 | 0.000689 | 0.025438 |
| Pulp 50°C | Wang-Singh | 0.801374 | 0.011909 | 0.106877 |
| Peel 65°C | Newton | 0.844307 | 0.00794 | 0.086596 |
| Peel 65°C | Page | 0.992448 | 0.000409 | 0.019072 |
| Peel 65°C | Modified Page | -0.33389 | 0.072277 | 0.253469 |
| Peel 65°C | Henderson & Pabis | 0.85166 | 0.008038 | 0.084527 |
| Peel 65°C | Logarithmic | 0.98259 | 0.001006 | 0.028958 |
| Peel 65°C | Two-Term | 0.999058 | 5.83E-05 | 0.006736 |
| Peel 65°C | Two-Term Exp | 0.999058 | 5.45E-05 | 0.006737 |
| Peel 65°C | Wang-Singh | 0.333167 | 0.036133 | 0.179215 |
| Pulp 65°C | Newton | 0.983233 | 0.001481 | 0.037709 |
| Pulp 65°C | Page | 0.992782 | 0.000665 | 0.024741 |
| Pulp 65°C | Modified Page | 0.992782 | 0.000665 | 0.024741 |
| Pulp 65°C | Henderson & Pabis | 0.986208 | 0.001271 | 0.0342 |
| Pulp 65°C | Logarithmic | 0.987162 | 0.001237 | 0.032996 |
| Pulp 65°C | Two-Term | 0.986208 | 0.001392 | 0.0342 |
| Pulp 65°C | Two-Term Exp | 0.986737 | 0.001278 | 0.033538 |
| Pulp 65°C | Wang-Singh | 0.992782 | 0.000665 | 0.024741 |
| Peel 75°C | Newton | 0.913469 | 0.004527 | 0.065274 |
| Peel 75°C | Page | 0.98354 | 0.000919 | 0.028469 |
| Peel 75°C | Modified Page | |  |  |
| Peel 75°C | Henderson & Pabis | 0.913662 | 0.004818 | 0.065201 |
| Peel 75°C | Logarithmic | 0.996935 | 0.000183 | 0.012284 |
| Peel 75°C | Two-Term | 0.998945 | 6.79E-05 | 0.007207 |
| Peel 75°C | Two-Term Exp | 0.913469 | 0.005174 | 0.065274 |
| Peel 75°C | Wang-Singh | 0.047039 | 0.053179 | 0.216616 |
| Pulp 75°C | Newton | 0.963347 | 0.00279 | 0.051607 |
| Pulp 75°C | Page | 0.964377 | 0.002847 | 0.050876 |
| Pulp 75°C | Modified Page | -0.22243 | 0.097705 | 0.298032 |
| Pulp 75°C | Henderson & Pabis | 0.965518 | 0.002756 | 0.050055 |
| Pulp 75°C | Logarithmic | 0.980562 | 0.001635 | 0.037582 |
| Pulp 75°C | Two-Term | 0.981629 | 0.001631 | 0.036536 |
| Pulp 75°C | Two-Term Exp | 0.963347 | 0.003084 | 0.051607 |
| Pulp 75°C | Wang-Singh | 0.837751 | 0.012968 | 0.108578 |

The ANOVA table presents the following parameters:

**DF (Degrees of Freedom):** The degrees of freedom corresponding to each model term.

**Seq SS (Sequential Sum of Squares):** The variance explained by each term when introduced sequentially into the model.

**Adj SS (Adjusted Sum of Squares):** The variance attributed to a specific term, adjusted to account for all other terms present in the model.

**Adj MS (Adjusted Mean Square):** Calculated as the ratio of Adj SS to DF (Adj SS / DF), representing the mean variance for each term.

**F-value:** The test statistic that compares the variance of each term against the residual error variance. A larger F-value indicates a higher probability that the term is significant.

**p-value:** Indicates the probability that the observed result occurred by random chance. Values of *p* < 0.05 are considered indicative of statistical significance

| Table S3. Analysis of Variance for TPC – Peel (3-way ANOVA) | | | | | | |
| --- | --- | --- | --- | --- | --- | --- |
| Source | **DF** | **Seq SS** | **Adj SS** | **Adj MS** | **F-value** | **P-value** |
| Moisture | 2 | 1.7288 | 1.7288 | 0.8644 | 7.48 | 0.002 |
| Temperature | 2 | 67.2204 | 67.2204 | 33.6102 | 290.78 | <0.001 |
| Process | 1 | 31.9596 | 31.9596 | 31.9596 | 276.50 | <0.001 |
| Moisture × Temperature | 4 | 1.0732 | 1.0732 | 0.2683 | 2.32 | 0.075 |
| Temperature × Process | 2 | 15.3759 | 15.3759 | 7.6879 | 66.51 | <0.001 |
| Moisture × Process | 2 | 0.1945 | 0.1945 | 0.0972 | 0.84 | 0.439 |
| Moisture × Temperature × Process | 4 | 1.2717 | 1.2717 | 0.3179 | 2.75 | 0.043 |

| Table S4. Significance groups of moisture–temperature–process for TPC-Peel | | | |
| --- | --- | --- | --- |
| Moisture (%) | **Temperature (°C)** | **Process** | **Grouping -Peel** |
| 5 | 75 | Oven | a |
| 10 | 75 | Oven | a |
| 15 | 75 | Oven | a,b |
| 5 | 65 | Oven | b |
| 10 | 65 | Oven | b |
| 15 | 65 | Oven | b |
| 5 | 75 | Ultrasound | c |
| 10 | 75 | Ultrasound | c,d |
| 5 | 65 | Ultrasound | c,d |
| 15 | 65 | Ultrasound | c,d,e |
| 5 | 50 | Oven | c,d,e,f |
| 15 | 75 | Ultrasound | d,e,f,g |
| 15 | 50 | Oven | e,f,g |
| 10 | 50 | Ultrasound | f,g |
| 5 | 50 | Ultrasound | f,g |
| 15 | 50 | Ultrasound | f,g |
| 10 | 50 | Oven | g |
| *Combinations that share at least one letter are not significantly different, whereas those with no letters in common differ significantly (p<0.05, Tukey’s, HSD), the letter ‘a’ is assigned to the group(s) with the highest mean value(s), so treatments carrying ‘a’ represent the statistically highest responses in the dataset* | | | |

| Table S5. Analysis of Variance for TPC – Pulp (3-way ANOVA) | | | | | | |
| --- | --- | --- | --- | --- | --- | --- |
| Source | **DF** | **Seq SS** | **Adj SS** | **Adj MS** | **F** | **P** |
| Moisture | 2 | 1.3492 | 1.3492 | 0.6746 | 19.53 | <0.001 |
| Temperature | 2 | 83.9810 | 83.9810 | 41.9905 | 1215.60 | <0.001 |
| Process | 1 | 0.9560 | 0.9560 | 0.9560 | 27.68 | <0.001 |
| Moisture × Temperature | 4 | 0.6649 | 0.6649 | 0.1662 | 4.81 | 0.003 |
| Moisture × Process | 2 | 1.8811 | 1.8811 | 0.9405 | 27.23 | <0.001 |
| Temperature × Process | 2 | 16.5269 | 16.5269 | 8.2635 | 239.22 | <0.001 |
| Moisture × Temperature × Process | 4 | 1.6527 | 1.6527 | 0.4132 | 11.96 | <0.001 |

| Table S6. Significance groups of moisture–temperature–process for TPC - Pulp | | | |
| --- | --- | --- | --- |
| Moisture (%) | **Temperature (°C)** | **Process** | **Grouping -Peel** |
| 10 | 75 | Oven | a |
| 5 | 75 | Oven | a |
| 15 | 75 | Oven | b |
| 5 | 75 | Ultrasound | c |
| 10 | 65 | Ultrasound | c |
| 10 | 75 | Ultrasound | c |
| 15 | 75 | Ultrasound | c,d |
| 15 | 65 | Ultrasound | c,d |
| 5 | 65 | Ultrasound | d |
| 5 | 65 | Oven | d |
| 10 | 65 | Oven | e |
| 15 | 65 | Oven | e,f |
| 10 | 50 | Oven | c,f |
| 5 | 50 | Oven | f,g |
| 10 | 50 | Ultrasound | f,g |
| 15 | 50 | Oven | f,g |
| 15 | 50 | Ultrasound | f,g |
| 5 | 50 | Ultrasound | g |
| *Combinations that share at least one letter are not significantly different, whereas those with no letters in common differ significantly (p<0.05, Tukey’s, HSD), the letter ‘a’ is assigned to the group(s) with the highest mean value(s), so treatments carrying ‘a’ represent the statistically highest responses in the dataset* | | | |

Moisture %

| Time (min) | Pulp 50 US |
| --- | --- |
| 0 | 88.1075 |
| 20 | 80.9781 |
| 40 | 72.8605 |
| 60 | 61.9193 |
| 80 | 53.331 |
| 100 | 44.3193 |
| 120 | 40.8605 |
| 140 | 31.0958 |
| 160 | 23.4487 |
| 180 | 20.5075 |
| 200 | 16.3899 |

| Time (min) | Peel 50 US |
| --- | --- |
| 0 | 84.1 |
| 20 | 46.2053 |
| 40 | 23.2388 |
| 60 | 7.44928 |
| 80 | 1.70766 |

Moisture %

| Time (min) | Pulp 65 US |
| --- | --- |
| 0 | 88.1075 |
| 20 | 78.1771 |
| 40 | 67.667 |
| 60 | 46.5696 |
| 80 | 35.8278 |
| 100 | 23.463 |
| 120 | 18.0534 |
| 140 | 14.1894 |
| 160 | 12.6438 |
| 180 | 10.3254 |

| Time (min) | Peel 65 US |
| --- | --- |
| 0 | 84.1 |
| 5 | 56.9281 |
| 10 | 50.2275 |
| 15 | 40.9854 |
| 20 | 34.0538 |
| 25 | 24.8116 |
| 30 | 19.2664 |
| 35 | 11.8726 |
| 40 | 10.0242 |
| 45 | 7.71368 |
| 50 | 5.86525 |

Moisture %

| Time (min) | Pulp 75 US |
| --- | --- |
| 0 | 88.1075 |
| 20 | 58.9044 |
| 40 | 31.2481 |
| 60 | 23.1231 |
| 80 | 21.7169 |
| 100 | 19.8419 |
| 120 | 16.5606 |
| 140 | 8.74814 |

| Time (min) | Peel 75 US |
| --- | --- |
| 0 | 84.1 |
| 2 | 53.8638 |
| 4 | 24.3362 |
| 6 | 16.8559 |
| 8 | 9.69055 |
| 10 | 6.14724 |
| 12 | 1.42283 |

**Figure S6**. Moisture loss for 50,65,75°C US pre-treatment for peel and pulp

| Table S7. Analysis of Variance for DPPH %Inhibition – Peel (3-way ANOVA) | | | | | |  |
| --- | --- | --- | --- | --- | --- | --- |
| Source | **DF** | **Seq SS** | **Adj SS** | **Adj MS** | **F** | **P** |
| Moisture | 2 | 679.81 | 679.81 | 339.90 | 21.97 | <0.001 |
| Temperature | 2 | 230.00 | 230.00 | 115.00 | 7.43 | 0.002 |
| Process | 1 | 9176.51 | 9176.51 | 9176.51 | 593.02 | <0.001 |
| Moisture × Temperature | 4 | 283.95 | 283.95 | 70.99 | 4.59 | 0.004 |
| Moisture × Process | 2 | 456.84 | 456.84 | 228.42 | 14.76 | <0.001 |
| Temperature × Process | 2 | 489.82 | 489.82 | 244.91 | 15.83 | <0.001 |
| Moisture × Temperature × Process | 4 | 332.32 | 332.32 | 83.08 | 5.37 | 0.002 |

| Table S8. Significance groups of moisture–temperature–process for DPPH % inhibition -Peel | | | |
| --- | --- | --- | --- |
| Moisture (%) | **Temperature (°C)** | **Process** | **Grouping -Peel** |
| 10 | 75 | Oven | a |
| 5 | 65 | Oven | a |
| 15 | 75 | Oven | a |
| 5 | 75 | Oven | a |
| 10 | 65 | Oven | a |
| 10 | 50 | Oven | a |
| 15 | 65 | Oven | a |
| 5 | 50 | Oven | a,b |
| 15 | 50 | Oven | a,b |
| 5 | 50 | Ultrasound | b,c |
| 5 | 75 | Ultrasound | b,c |
| 5 | 65 | Ultrasound | c |
| 15 | 65 | Ultrasound | c,d |
| 10 | 65 | Ultrasound | c,d |
| 10 | 50 | Ultrasound | d,e |
| 10 | 75 | Ultrasound | d,e |
| 15 | 50 | Ultrasound | e |
| 15 | 75 | Ultrasound | e |

*Combinations that share at least one letter are not significantly different, whereas those with no letters in common differ significantly (p<0.05, Tukey’s, HSD), the letter ‘a’ is assigned to the group(s) with the highest mean value(s), so treatments carrying ‘a’ represent the statistically highest responses in the dataset*

| Table S9. Analysis of Variance for DPPH %Inhibition – Pulp (3-way ANOVA) | | | | | | |
| --- | --- | --- | --- | --- | --- | --- |
| Source | **DF** | **Seq SS** | **Adj SS** | **Adj MS** | **F** | **P** |
| Moisture | 2 | 521.47 | 521.47 | 260.73 | 24.25 | <0.001 |
| Temperature | 2 | 4572.60 | 4572.60 | 2286.30 | 212.67 | <0.001 |
| Process | 1 | 150.30 | 150.30 | 150.30 | 13.98 | 0.001 |
| Moisture × Temperature | 4 | 326.51 | 326.51 | 81.63 | 7.59 | <0.001 |
| Moisture × Process | 2 | 33.45 | 33.45 | 16.72 | 1.56 | 0.225 |
| Temperature × Process | 2 | 344.35 | 344.35 | 172.18 | 16.02 | <0.001 |
| Moisture × Temperature × Process | 4 | 220.34 | 220.34 | 55.09 | 5.12 | 0.002 |

| Table S10. Significance groups of moisture–temperature–process for DPPH % inhibition-Pulp | | | |
| --- | --- | --- | --- |
| Moisture (%) | **Temperature (°C)** | **Process** | **Grouping -Pulp** |
| 10 | 75 | Ultrasound | a |
| 15 | 75 | Ultrasound | a,b |
| 5 | 75 | Ultrasound | a,b |
| 5 | 75 | Oven | a,b |
| 15 | 75 | Oven | a,b |
| 10 | 75 | Oven | a,b,c |
| 5 | 50 | Oven | b,c,d |
| 10 | 50 | Oven | c,d,e |
| 5 | 65 | Oven | c,d,e |
| 5 | 50 | Ultrasound | c,d,e,f |
| 10 | 50 | Ultrasound | d,e,f |
| 5 | 65 | Ultrasound | e,f |
| 15 | 50 | Ultrasound | e,f |
| 10 | 65 | Oven | e,f |
| 15 | 50 | Oven | e,f |
| 15 | 65 | Oven | e,f |
| 10 | 65 | Ultrasound | f |
| 15 | 65 | Ultrasound | g |
| *Combinations that share at least one letter are not significantly different, whereas those with no letters in common differ significantly (p<0.05, Tukey’s, HSD), the letter ‘a’ is assigned to the group(s) with the highest mean value(s), so treatments carrying ‘a’ represent the statistically highest responses in the dataset* | | | |

IC50 mg/mL

|  | Peel 5% | Pulp 5% | Peel 10% | Pulp 10% | Peel 15% | Pulp 15% |
| --- | --- | --- | --- | --- | --- | --- |
| 40°C Oven | 0.4596 | 0.5979 | 0.5114 | 0.6647 | 0.6254 | 0.6647 |
| 50°C Oven | 0.4621 | 2.2863 | 0.7755 | 2.7349 | 1.2655 | 3.1445 |
| 65°C Oven | 0.4539 | 2.1556 | 2.0403 | 3.0276 | 3.1448 | 3.4267 |
| 75°C Oven | 0.5722 | 1.4712 | 0.6274 | 1.6563 | 0.6648 | 1.8728 |
| 50°C US | 0.9469 | 2.3806 | 1.2518 | 2.78 | 1.8768 | 3.7194 |
| 65°C US | 0.6999 | 0.9324 | 0.8528 | 1.092 | 1.21 | 1.612 |
| 75°C US | 0.6568 | 0.8258 | 0.82884 | 1.0967 | 0.9975 | 1.34 |

**Figure S7**. Figure 5. IC50 values for different temperatures (40°C, 50°C, 65°C, and 75 °C) for peel and pulp, for 3 levels of moisture (5%,10%,15%) without and with pre-treatment US,

**Table S11.** Experimental Design and Results TPC (mg GE/g DW) and TFC (mg QE/g DW)

| **Run No.** | **X1** | **X2** | **X3** | **Peel** | | **Pulp** | |
| --- | --- | --- | --- | --- | --- | --- | --- |
|  |  |  |  | **TPC_pred_** | **TPC_exp_** | **TPC_pred_** | **TPC_exp_** |
| 1 | 17.5 | 50 | 0.05 | 8.786 | 9.896 | 3.829 | 4.205 |
| 2 | 17.5 | 75 | 0.05 | 9.682 | 9.162 | 2.875 | 3.207 |
| 3 | 30 | 50 | 0.05 | 9.986 | 9.830 | 4.728 | 4.878 |
| 4 | 30 | 75 | 0.033 | 6.550 | 6.612 | 3.307 | 3.250 |
| 5 | 5 | 25 | 0.1 | 10.466 | 10.746 | 4.162 | 4.623 |
| 6 | 17.5 | 50 | 0.05 | 9.674 | 9.896 | 3.661 | 4.205 |
| 7 | 17.5 | 50 | 0.05 | 9.742 | 9.896 | 4.094 | 4.205 |
| 8 | 5 | 75 | 0.1 | 10.895 | 10.053 | 2.856 | 3.229 |
| 9 | 5 | 75 | 0.033 | 6.927 | 7.056 | 1.675 | 1.421 |
| 10 | 17.5 | 50 | 0.033 | 7.975 | 6.716 | 2.643 | 2.512 |
| 11 | 30 | 25 | 0.033 | 7.563 | 6.975 | 3.553 | 3.996 |
| 12 | 17.5 | 50 | 0.1 | 10.763 | 10.664 | 5.785 | 5.446 |
| 13 | 17.5 | 50 | 0.05 | 9.929 | 9.896 | 3.85 | 4.21 |
| 14 | 5 | 50 | 0.05 | 10.318 | 10.200 | 4.005 | 3.179 |
| 15 | 60 | 75 | 0.1 | 11.057 | 10.608 | 4.816 | 5.265 |
| 16 | 30 | 25 | 0.1 | 11.461 | 10.513 | 6.607 | 6.163 |
| 17 | 17.5 | 25 | 0.05 | 9.670 | 9.587 | 4.844 | 4.174 |
| 18 | 30 | 75 | 0.1 | 8.741 | 9.735 | 5.745 | 5.002 |
| 19 | 5 | 25 | 0.033 | 7.255 | 7.333 | 2.519 | 2.399 |
| 20 | 17.5 | 50 | 0.05 | 9.857 | 9.896 | 3.789 | 4.205 |
| 21 | 60 | 50 | 0.05 | 11.506 | 10.643 | 5.810 | 5.052 |
| 22 | 60 | 25 | 0.1 | 11.232 | 11.490 | 5.842 | 6.147 |
| 23 | 60 | 25 | 0.033 | 7.223 | 7.802 | 3.999 | 4.048 |
| 24 | 60 | 75 | 0.033 | 7.189 | 7.335 | 3.500 | 3.581 |

*X_1_ = Time (min); X_2_ = Mass Ratio (g/mL); X_3_ = Water (%). p-values significance a= p < 0.05, b = p < 0.01, and c = p < 0.001, respectively.*

| **Table S12**. Regression coefficients and p-values for the second-order polynomial models of TPC and TFC for peel and pulp. | | | | |
| --- | --- | --- | --- | --- |
|  | **TPC Peel** | **TFC Peel** | **TPC Pulp** | **TFC Pulp** |
| **Term** | **Coeff.** | **Coeff.** | **Coeff.** | **Coeff.** |
| Constant | -0.20000 | 0.37130 ^b^ | 1.5913 | -0.43570 |
| X₁ | -0.00271 | 0.03787 ^a^ | 0.15251 ^c^ | 0.00857 |
| X₂ | 268.73^c^ | 42.2600 | -49.4500 | 60.9200 ^b^ |
| X₃ | 0.02763 | -0.02663 ^a^ | 0.06945 | -0.03734 ^b^ |
| X₁² | -6.100e-6 | -3.720e-4 ^b^ | -0.00197 ^c^ | -1.133e-4 |
| X₂² | -1672.20 ^c^ | -249.60 | 582.40 ^a^ | -424.20 ^b^ |
| X₃² | -3.646e-4 | 1.639e-4 ^b^ | -9.090e-4 ^a^ | 3.309e-4 ^a^ |
| X₁X₂ | 0.15530 | 0.01013 | 0.01240 | 0.04152 |
| X₁X₃ | -6.650e-5 | -2.396e-5 | 1.261e-4 | 3.370e-5 |
| X₂X₃ | 0.09390 | 0.06301 | -0.03671 | 0.04272 |
| **R²** | 0.953 | 0.903 | 0.873 | 0.868 |
| **R²(adj)** | 0.910 | 0.841 | 0.791 | 0.785 |
| **Standard Error** | 0.469 | 0.187 | 0.561 | 0.121 |

| Table S13: ANOVA analysis for model of TPC Peel | | | | | | |
| --- | --- | --- | --- | --- | --- | --- |
| Source | DF | Seq SS | Adj SS | Adj MS | F | P |
| Blocks | 1 | 0.0541 | 0.1002 | 0.10023 | 0.46 | 0.514 |
| Regression | 9 | 49.0318 | 49.0318 | 5.44798 | 24.76 | <0.001 |
| Linear | 3 | 37.8448 | 973.53% | 3.2451 | 14.75 | <0.001 |
| Time | 1 | 0.1107 | 0.0918 | 0.09183 | 0.42 | 0.532 |
| Mass Ratio | 1 | 37.6637 | 7.2913 | 7.29128 | 33.13 | <0.001 |
| Solvent Ratio | 1 | 0.0704 | 0.1325 | 0.13245 | 0.6 | 0.454 |
| Square | 3 | 10.9182 | 10.0687 | 3.35623 | 15.25 | <0.001 |
| Time*Time | 1 | 2.8132 | 0.095 | 0.09504 | 0.43 | 0.525 |
| Mass Ratio*Mass Ratio | 1 | 7.8679 | 5.6651 | 5.66507 | 25.74 | <0.001 |
| Solvent Ratio*Solvent Ratio | 1 | 0.2371 | 0.2253 | 0.2253 | 1.02 | 0.333 |
| Interaction | 3 | 0.2688 | 0.2688 | 0.08961 | 0.41 | 0.751 |
| Time*Mass Ratio | 1 | 0.176 | 0.1784 | 0.17839 | 0.81 | 0.387 |
| Time*Solvent Ratio | 1 | 0.0148 | 0.0179 | 0.01788 | 0.08 | 0.781 |
| Mass Ratio*Solvent Ratio | 1 | 0.078 | 0.078 | 0.07798 | 0.35 | 0.564 |
| Residual Error | 11 | 2.4206 | 2.4206 | 0.22006 |  |  |
| Lack-of-Fit | 7 | 1.5585 | 1.5585 | 0.22264 | 1.03 | 0.519 |
| Pure Error | 4 | 0.8621 | 0.8621 | 0.21553 |  |  |
| Total | 21 | 51.5065 |  |  |  |  |

| Table S14: ANOVA analysis for model of TPC Pulp | | | | | | |
| --- | --- | --- | --- | --- | --- | --- |
| Source | DF | Seq SS | Adj SS | Adj MS | F | P |
| Blocks | 1 | 1.3787 | 0.0086 | 0.00864 | 0.11 | 0.743 |
| Regression | 9 | 28.5463 | 28.5463 | 3.17181 | 41.98 | <0.001 |
| Linear | 3 | 27.4924 | 19.462 | 6.48734 | 85.86 | <0.001 |
| Time | 1 | 10.5253 | 8.7838 | 8.78382 | 116.26 | <0.001 |
| Mass Ratio | 1 | 13.4785 | 6.8915 | 6.8915 | 91.21 | <0.001 |
| Solvent Ratio | 1 | 3.4886 | 2.2279 | 2.22788 | 29.49 | <0.001 |
| Square | 3 | 0.9841 | 0.9721 | 0.32403 | 4.29 | 0.039 |
| Time×Time | 1 | 0.4307 | 0.1059 | 0.10589 | 1.4 | 0.267 |
| Mass Ratio×Mass Ratio | 1 | 0.0466 | 0.3106 | 0.31059 | 4.11 | 0.073 |
| Solvent Ratio×Solvent Ratio | 1 | 0.5068 | 0.4544 | 0.45438 | 6.01 | 0.037 |
| Interaction | 3 | 0.0698 | 0.0698 | 0.02327 | 0.31 | 0.819 |
| Time×Mass Ratio | 1 | 0.0018 | 0.0016 | 0.00162 | 0.02 | 0.887 |
| Time×Solvent Ratio | 1 | 0.0549 | 0.0548 | 0.05485 | 0.73 | 0.416 |
| Mass Ratio×Solvent Ratio | 1 | 0.0132 | 0.0132 | 0.01317 | 0.17 | 0.686 |
| Residual Error | 9 | 0.68 | 0.68 | 0.07556 |  |  |
| Lack-of-Fit | 5 | 0.5807 | 0.5807 | 0.11613 | 4.68 | 0.08 |
| Pure Error | 4 | 0.0994 | 0.0994 | 0.02484 |  |  |
| Total | 19 | 30.605 |  |  |  |  |

| Table S15: ANOVA for model of TFC Peel | | | | | | |
| --- | --- | --- | --- | --- | --- | --- |
| Source | **DF** | **Seq SS** | **Adj SS** | **Adj MS** | **F-value** | **P-value** |
| Blocks | 1 | 0.4355 | 0.15227 | 0.15227 | 38.92 | <0.001 |
| Regression | 9 | 2.24805 | 2.24805 | 0.24978 | 63.84 | <0.001 |
| Linear | 3 | 2.0287 | 0.15314 | 0.05105 | 13.05 | 0.002 |
| Time | 1 | 0.73127 | 0.0291 | 0.0291 | 7.44 | 0.026 |
| Mass Ratio | 1 | 1.21822 | 0.0071 | 0.0071 | 1.81 | 0.215 |
| Solvent Ratio | 1 | 0.07921 | 0.03835 | 0.03835 | 9.8 | 0.014 |
| Square | 3 | 0.16664 | 0.16092 | 0.05364 | 13.71 | 0.002 |
| Time×Time | 1 | 0.02102 | 0.10816 | 0.10816 | 27.64 | 0.001 |
| Mass Ratio×Mass Ratio |  | 0.09931 | 0.00086 | 0.00086 | 0.22 | 0.653 |
| Solvent Ratio×Solvent Ratio | 1 | 0.04631 | 0.04669 | 0.04669 | 11.93 | 0.009 |
| Interaction | 3 | 0.0527 | 0.0527 | 0.01757 | 4.49 | 0.04 |
| Time×Mass Ratio | 1 | 0.0131 | 0.01335 | 0.01335 | 3.41 | 0.102 |
| Time×Solvent Ratio | 1 | 0.03566 | 0.01807 | 0.01807 | 4.62 | 0.064 |
| Mass Ratio×Solvent Ratio | 1 | 0.00395 | 0.00395 | 0.00395 | 1.01 | 0.345 |
| Residual Error | 8 | 0.0313 | 0.0313 | 0.00391 |  |  |
| Lack-of-Fit | 4 | 0.02305 | 0.02305 | 0.00576 | 2.79 | 0.172 |
| Pure Error | 4 | 0.00825 | 0.00825 | 0.00206 |  |  |
| Total | 18 | 2.71485 |  |  |  |  |

| Table S16: ANOVA for model of TFC Pulp | | | | | | |
| --- | --- | --- | --- | --- | --- | --- |
| Source | DF | Seq SS | Adj SS | Adj MS | F | P |
| Blocks | 1 | 0.11909 | 0.000001 | 1E-06 | 0 | 0.993 |
| Regression | 9 | 0.87826 | 0.87826 | 0.09758 | 10.79 | 0.001 |
| Linear | 3 | 0.58751 | 0.460571 | 0.15352 | 16.97 | <0.001 |
| Time | 1 | 0.00808 | 0.072998 | 0.073 | 8.07 | 0.019 |
| Mass Ratio | 1 | 0.56416 | 0.443423 | 0.44342 | 49.03 | <0.001 |
| Solvent Ratio | 1 | 0.01527 | 0.003003 | 0.003 | 0.33 | 0.579 |
| Square | 3 | 0.26082 | 0.2145 | 0.0715 | 7.91 | 0.007 |
| Time×Time | 1 | 0.03315 | 0.000735 | 0.00074 | 0.08 | 0.782 |
| Mass Ratio×Mass Ratio | 1 | 0.14301 | 0.174949 | 0.17495 | 19.34 | 0.002 |
| Solvent Ratio×Solvent Ratio | 1 | 0.08466 | 0.064295 | 0.0643 | 7.11 | 0.026 |
| Interaction | 3 | 0.02993 | 0.029927 | 0.00998 | 1.1 | 0.397 |
| Time×Mass Ratio | 1 | 0.0138 | 0.011125 | 0.01113 | 1.23 | 0.296 |
| Time×Solvent Ratio | 1 | 0.0032 | 0.003812 | 0.00381 | 0.42 | 0.532 |
| Mass Ratio×Solvent Ratio | 1 | 0.01293 | 0.012933 | 0.01293 | 1.43 | 0.262 |
| Residual Error | 9 | 0.0814 | 0.081403 | 0.00905 |  |  |
| Lack-of-Fit | 5 | 0.06826 | 0.068263 | 0.01365 | 4.16 | 0.096 |
| Pure Error | 4 | 0.01314 | 0.01314 | 0.00329 |  |  |
| Total | 19 | 1.07875 |  |  |  |  |

| Table S18. Experimental and predicted values from externals validation experiments for peel & pulp | | | | | | | | |
| --- | --- | --- | --- | --- | --- | --- | --- | --- |
|  | **Peel** | | | | **Pulp** | | | |
| Run No. | **TPC_exp_** | **TPC_pred_** | **TFC_exp_** | **TFCp_red_** | **TPC_exp_** | **TPC_pred_** | **TFC_exp_** | **TFC_pre_d** |
| 1 | 10.49 ± 0.48 | 10.70 | 1.86 ± 0.04 | 1.65 | 3.74 ± 0.08 | 3.30 | 0.79 ± 0.17 | 0.91 |
| 2 | 9.00 ± 0.25 | 8.43 | 1.69 ± 0.04 | 1.42 | 3.34 ± 0.25 | 3.86 | 0.81 ± 0.08 | 0.58 |
| 3 | 12.20 ± 0.37 | 11.56 | 2.21 ± 0.23 | 2.18 | 5.68 ± 0.23 | 5.03 | 1.03 ± 0.08 | 1.12 |
| 4 | 9.03 ± 0.21 | 8.32 | 2.05 ± 0.15 | 1.69 | 3.74 ± 0.15 | 4.40 | 0.76 ± 0.20 | 0.73 |
| 5 | 12.73 ± 0.72 | 11.70 | 2.89 ± 0.33 | 2.55 | 5.59 ± 0.22 | 5.70 | 1.16 ± 0.15 | 1.30 |

| Table S17. External validation of the RSM models for TPC and TFC in peel and pulp | | | |
| --- | --- | --- | --- |
| Run No. | Time(min) | Ratio Sample/Solvent (g/mL) | EtOH % |
| 1 | 10 | 1:15 | 55 |
| 2 | 21 | 1:20 | 40 |
| 3 | 21 | 1:12.5 | 60 |
| 4 | 45 | 1:20 | 35 |
| 5 | 45 | 1:12.5 | 65 |

The predictive performance of the fitted second-order polynomial models was assessed against an independent set of validation experiments, the results of which are reported in Table S19. Five metrics were calculated, as defined in Eqs (S1)–(S5).

$$\mathrm{MAE}=\frac{1}{n}\sum_{i=1}^{n} \left| y_{i}-\hat{y}_{i} \right|$$

(S1)

$$\mathrm{RMSE}=\sqrt{\frac{1}{n}\sum_{i=1}^{n} \left( y_{i}-\hat{y}_{i} \right)^{2}}$$

(S2)

$$\mathrm{MAPE}=\frac{100}{n}\sum_{i=1}^{n} \left| \frac{y_{i}-\hat{y}_{i}}{y_{i}} \right|$$

(S3)

$$R^{2}=1-\frac{\sum_{i=1}^{n} \left( y_{i}-\hat{y}_{i} \right)^{2}}{\sum_{i=1}^{n} \left( y_{i}-\bar{y} \right)^{2}}$$

(S4)

$$\mathrm{Bias}=\frac{1}{n}\sum_{i=1}^{n} \left( \hat{y}_{i}-y_{i} \right)$$

(S5)

where:

*yᵢ*: observed (experimentally measured) value of the response at validation point i, expressed in the units of the response (mg GAE g⁻¹ DW for TPC; mg QE g⁻¹ DW for TFC);

*ŷᵢ*: value predicted at validation point i by the fitted second-order polynomial model, in the same units as the observed response.

*ȳ*: arithmetic mean of the observed values over all validation points, in the same units as the observed response.

*n*: number of independent validation points constituting the external validation set (dimensionless);

*i*: index of the validation point, running from 1 to n (dimensionless).

MAE (mean absolute error) and RMSE (root mean square error) are both expressed in the units of the response and are therefore directly comparable with the measured values. RMSE weights large deviations more heavily than MAE, so that a value of RMSE appreciably greater than MAE indicates that the error is dominated by a small number of larger departures rather than being uniformly distributed.

MAPE (mean absolute percentage error) expresses the deviation relative to the observed value and is therefore dimensionless, which permits comparison between responses of differing magnitude. It is undefined for observations equal to zero and becomes disproportionately large for small observed values.

R² is calculated on the external validation set relative to the mean of the observed validation values. It is therefore not equivalent to the coefficient of determination reported in Table 5, which is calculated on the same observations used to estimate the regression coefficients. Because the external R² is not constrained to the interval [0, 1], a negative value is possible and would indicate that the model predicts the validation data less accurately than their own mean.

Bias is defined here as predicted minus observed, as given in Eq. (S5). On this convention a positive value denotes systematic over-prediction by the model and a negative value systematic under-prediction. A bias small in magnitude relative to the corresponding RMSE indicates that the residual error is predominantly random rather than systematic.

| Table S19: Statistical metrics for the external validation of the predictive response surface models. | | | | |
| --- | --- | --- | --- | --- |
|  | Peel TPC | Peel TFC | Pulp TPC | Pulp TFC |
| MAE | 0.126 | 0.049 | 0.095 | 0.025 |
| RMSE | 0.682 | 0.271 | 0.515 | 0.140 |
| MAPE | 5.892 | 11.643 | 11.642 | 8.138 |
| R^2^ | 0.8072 | 0.8997 | 0.7366 | 0.7763 |
| Bias | -0.109 | -0.049 | 0.008 | 0.004 |
